# TTK inhibitor OSU13 promotes immunotherapy responses by activating tumor STING

**DOI:** 10.1172/jci.insight.177523

**Published:** 2024-06-20

**Authors:** Vijaya Bharti, Amrendra Kumar, Yinchong Wang, Nikhil Roychowdhury, Daniel de Lima Bellan, Beimnet B. Kassaye, Reese Watkins, Marina Capece, Catherine G. Chung, Gerard Hilinski, Anna E. Vilgelm

**Affiliations:** 1 Department of Pathology,; 2 Pelotonia Institute for Immunooncology, and; 3 Comprehensive Cancer Center, Arthur G. James Cancer Hospital and Richard J. Solove Research Institute, The Ohio State University, Columbus, Ohio, USA.; 4 Molecular Cellular and Developmental Biology Graduate Program, The Ohio State University, Columbus, Ohio, USA.; 5 Department of Pathology and Dermatology and; 6 Drug Development Institute, Comprehensive Cancer Center and The James Cancer Hospital and Solove Research Institute, Columbus, Ohio, USA.

**Keywords:** Oncology, Cancer immunotherapy, Cell stress, Chemokines

## Abstract

TTK spindle assembly checkpoint kinase is an emerging cancer target. This preclinical study explored the antitumor mechanism of TTK inhibitor OSU13 to define a strategy for clinical development. We observed prominent antitumor activity of OSU13 in melanoma, colon and breast cancer cells, organoids derived from patients with melanoma, and mice bearing colon tumors associated with G_2_ cell cycle arrest, senescence, and apoptosis. OSU13-treated cells displayed DNA damage and micronuclei that triggered the cytosolic DNA-sensing cGAS/STING pathway. STING was required for the induction of several proteins involved in T cell recruitment and activity. Tumors from OSU13-treated mice showed an increased proportion of T and NK cells and evidence of PD-1/PD-L1 immune checkpoint activation. Combining a low-toxicity dose of OSU13 with anti–PD-1 checkpoint blockade resulted in prominent STING- and CD8^+^ T cell–dependent tumor inhibition and improved survival. These findings provide a rationale for utilizing TTK inhibitors in combination with immunotherapy in STING-proficient tumors.

## Abstract 2

A preclinical study of a small-molecule TTK inhibitor identifies potent proimmunogenic activity reliant on the induction of tumor-intrinsic cytosolic DNA-sensing pathway.

## Introduction

Monopolar spindle kinase 1 (MPS1, also known as TTK) is an essential component of the spindle assembly checkpoint (SAC). The SAC ensures proper chromosome segregation during mitosis and meiosis by halting division until sister chromatids make it to opposite ends of the dividing cell (1). Dysfunctional SAC may lead to the accumulation of chromosome segregation errors and DNA damage. Cancer cells divide frequently and, therefore, are sensitive to SAC inhibition. Drugs targeting essential components of the SAC, such as TTK, are promising emerging therapeutic tools in cancer treatment.

Several TTK inhibitors (TTKi) have been developed and tested in human trials (2–5), but none has been approved for clinical use to date. While TTKi show a clear effect on tumor cell growth and viability in vitro, the extent of their in vivo activity may not be sufficient to justify the development of single-agent TTKi therapeutics. For instance, in a mouse xenograft study, the TTKi CFI-402257 did not outperform single-agent carboplatin therapy (6). Similarly, S81694, which was tested as a single agent in adults with solid tumors, showed relatively mild toxicities but primarily induced disease stabilization (7). The study was terminated early, because it was decided to prioritize use of S81694 in patients along with additional cytotoxic agents. This decision was based on preclinical data that emerged during the trial that showed that TTK inhibition augments responses to microtubule poisons paclitaxel and docetaxel (8–10). Without functional SACs, microtubule poisons induced chromosomal segregation defects and death of tumor cells in preclinical models. A combination of TTKi BAY1217389 and paclitaxel was recently tested in a phase I clinical trial in solid tumors (11). However, severe hematological and other toxicities were observed in combination-treated patients. Other studies tested similar regimens of the dual TTK/PLK1 inhibitor BAL0891 as monotherapy and in combination with carboplatin or paclitaxel (ClinicalTrials.gov NCT05768932). Another TTKi, CFI-402257, was tested in combination with paclitaxel in patients with advanced HER2^–^ breast cancer (ClinicalTrials.gov NCT03568422). Interim reports from these studies indicate some evidence of efficacy; however, overall reported response rates are not high (about 11%–14%). Furthermore, there are toxicities reported, including fatigue, neutropenia, anemia, alopecia, diarrhea, and nausea (12–15). Overall, the clinical utility of paclitaxel and TTKi combinations remains to be demonstrated.

Aside from paclitaxel and other chemotherapies, there is an ongoing investigation of hormonal therapy in combination with TTKi. For instance, the ClinicalTrials.gov NCT02792465 study evaluated CFI-402257 as monotherapy and in combination with fulvestrant in a phase I trial in patients with advanced solid tumors, including HER2^–^ breast cancer. This study reported early signs of antitumor activity with a manageable safety profile, both as a monotherapy and in combination with fulvestrant in patients with ER^+^/HER2^–^ breast cancer who have failed CDK4/6 inhibitors (16). The follow-up study is currently ongoing (ClinicalTrials.gov NCT05251714).

OSU13 is a small-molecule inhibitor of TTK designed by computer-assisted docking analyses. Initial studies showed an antiproliferative effect of OSU13 in breast cancer cell lines (17) and in human multiple myeloma cells and xenografts (18). It has been shown to have high selectivity for TTK over almost 400 other human kinases in the kinome scan assay (17). Here, we focused on the mechanism of action of OSU13 in solid tumors, particularly its ability to enhance tumor immune recognition. We hypothesized that OSU13 would promote tumor infiltration by the immune cells based on our experience with other antiproliferative drugs. For example, inhibitors of cyclin-dependent kinases 4 and 6 (CDK4/6i) that are used to treat metastatic ER^+^ breast cancer can induce the production of T cell–attracting chemokines in tumors and promote T cell responses (19, 20). Similarly, a drug targeting the activity of a mitotic kinase, Aurora A, can facilitate immune cell infiltration into tumors by inducing the secretion of chemokine CCL5 from tumor cells. The release of immune cell–attracting chemokines results from senescence and the senescence-associated secretory phenotype (SASP) induced in tumor cells under prolonged drug-mediated cell cycle arrest and damage (21–23). Senescence is a type of stress response characterized by a loss of proliferative potential and the increased secretion of proinflammatory mediators, growth factors, stromal components, and other molecules that affect the local microenvironment (24, 25). This damage-associated proinflammatory response in tumors may promote their recognition by the immune system, which could be beneficial in the context of immunotherapy, a type of cancer treatment that enhances the activity of antitumor immune cells. This includes immune checkpoint blockade (ICB) therapy that targets inhibitory receptors on T cells or their ligands expressed in tissues (26). Unlike many other cancer treatments that only provide short-lived benefits, the effects of ICB can be long-lasting. However, many tumors are resistant to ICB because they are ignored by the immune system. Combining ICB with drugs promoting tumor immune recognition can overcome ICB resistance, which is an urgent and unmet clinical need (27). Our results provide strong rationale for clinical development of a combination treatment using OSU13 and ICB to enhance antitumor immunity.

## Results

**Figure 1:**
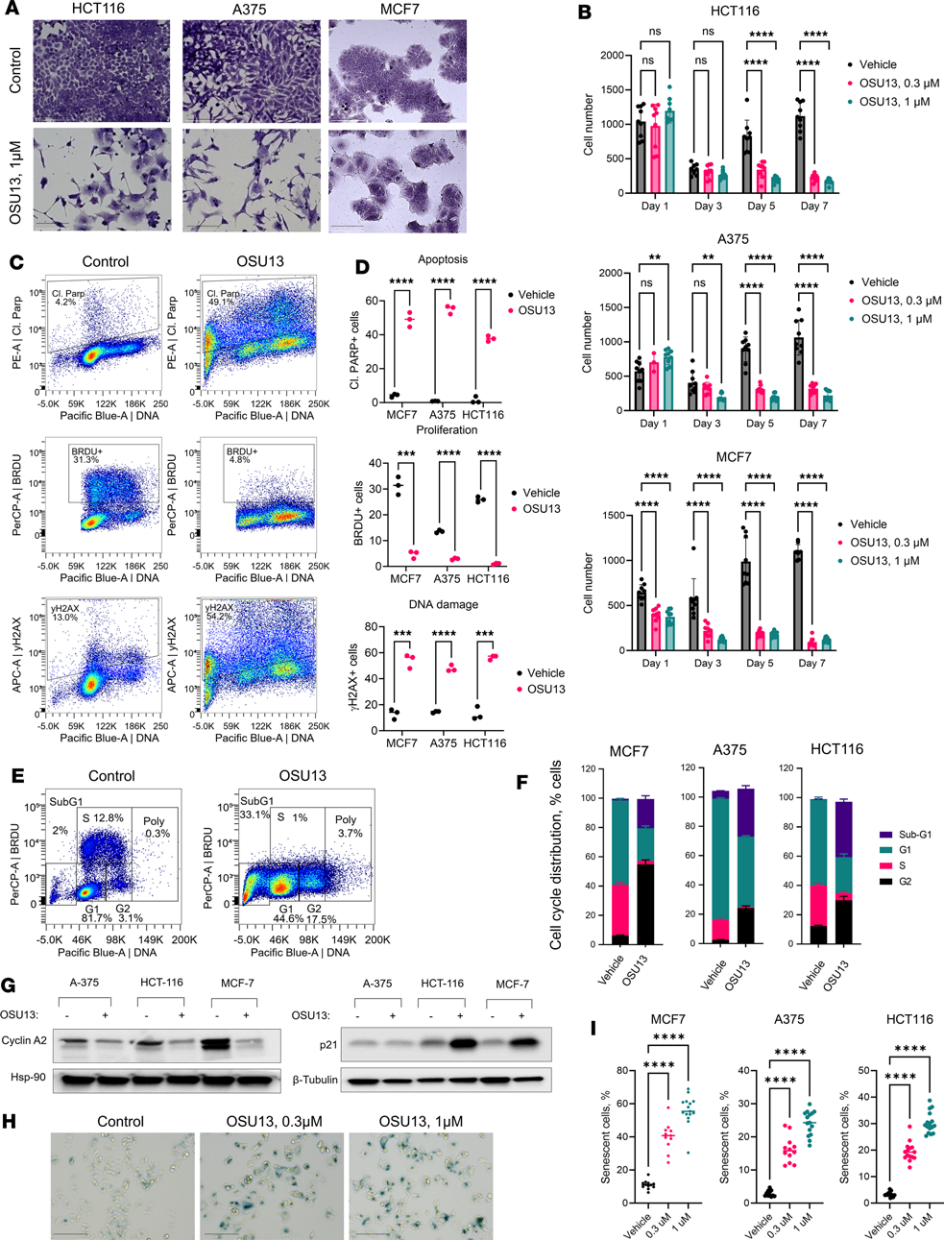
OSU13 inhibits tumor cells by inducing apoptosis, cell cycle arrest, and senescence. (**A**) Representative images of colon (HCT116), melanoma (A375), and breast (MCF7) tumor cells treated with vehicle or 1 μM OSU13 for 5 days and stained with crystal violet. Scale bar: 150 μm. (**B**) Cell numbers in indicated cultured cells treated with 0.3 or 1 μM OSU13 or vehicle. Cells were imaged at 1, 3, 5, and 7 days after treatment. *N* = 9 microscopic fields from 3 wells in a 6-well plate per condition. Statistics were performed using 2-way ANOVA with Dunnett’s post test. (**C**) Representative flow cytometry plots of MCF7 cells treated with vehicle control or 0.3 μM OSU13 for 5 days and stained with fluorescent antibodies recognizing cleaved PARP, BRDU, and γH2AX. BRDU was added to the cell culture media 4 hours before cell collection at the concentration of 25 μg/mL. (**D**) Quantified data from flow cytometry analysis shown in **C** in indicated cancer cell lines. *N* = 3 replicates per condition. Statistical analyses using multiple unpaired *t* tests. (**E**) Representative results of cell cycle phase gating in A375 cells analyzed as described in **C**. (**F**) Quantified cell cycle distribution data from analysis shown in **E** across 3 indicated cancer cell lines. *N* = 3 biological replicates per condition. (**G**) Western blot analysis of cell cycle–related proteins in A375, HCT-116, and MCF7 cells treated with 0.3 μM OSU13 or vehicle for 5 days. (**H**) Representative microphotographs of MCF7 cells treated for 3 days with 0.3 or 1 μM OSU13 or vehicle and stained for the activity of senescence-associated β-galactosidase (SA-β-Gal). Scale bar: 150 μm. (**I**) Percentages of SA-β-Gal^+^ cells quantified from the experiment shown in **H**. Three indicated cancer cell lines were used. *N* = 12–15 microscopic fields per condition (across 3 wells in a 6-well plate). Statistics using 1-way ANOVA with Dunnett’s multiple comparisons tests. **P < 0.01; ***P < 0.001; ****P < 0.0001.

The objective of this study was to investigate the antitumor efficacy of OSU13, a TTKi. To determine the direct antitumor activity of OSU13, we treated colon, melanoma, and breast cancer cells with various concentrations of OSU13 or vehicle for 1, 3, 5, and 7 days and counted the cells. There were significantly fewer cells after treatment with OSU13 compared with vehicle control, and this effect was dose and time dependent (Figure 1, A and B). To define the mechanism of tumor cell inhibition by OSU13, we employed a flow cytometry–based assay measuring apoptosis, replication, and DNA damage. Approximately half of the cells treated with OSU13 displayed cleavage of PARP, indicative of apoptosis (Figure 1, C and D). The remaining live cells failed to incorporate BRDU, indicating an arrested cell cycle (Figure 1, C and D). A more detailed analysis of cell cycle distribution revealed a decreased proportion of cells in G_1_ and S phases and an accumulation of cells in G_2_ (Figure 1, E and F). There was an increased proportion of cells with less than a 2n amount of DNA (sub-G_1_), which is a marker of apoptosis. Consistent with inhibition of the cell cycle, we observed decreased expression of the cell cycle protein cyclin A2 and induction of cell cycle arrest mediator p21 after OSU13 treatment (Figure 1G). Furthermore, many OSU13-treated cells had high levels of phosphorylated histone H2AX (γH2AX), a DNA damage marker (Figure 1, C and D). Persistent cell cycle arrest in conjunction with DNA damage is often observed in cells undergoing senescence. Accordingly, we detected a prominent induction of senescence-associated β-galactosidase activity after OSU13 treatment in all 3 tested cancer lines (Figure 1, H and I). Altogether, these results show that OSU13 induced DNA damage, G_2_ cell cycle arrest, senescence, and apoptosis in cancer cells.

Our next objective was to test if TTK is the main cell target of OSU13. Previously, our colleagues from The Ohio State University showed that OSU13 has high selectivity for TTK over almost 400 other human kinases in the cell-free kinome scan assay (18). Specifically, OSU13 half-maximal inhibitory concentration was shown to be the lowest for TTK (4.3 nM). The only other kinase that was targeted by OSU13 with a single-digit nM concentration was LRRK2 (7.5 nM). Therefore, we tested OSU13 in a LRRK2 NanoBRET assay to determine whether OSU13 can prevent the binding of an ATP analog to the kinase of interest (LRRK2) in mammalian cells. The receptor protein tyrosine kinase inhibitor CEP-701 was used as the positive control. Though our colleagues previously showed that OSU13 achieves an EC_50_ of 10 nM in a TTK NanoBRET in-cell target engagement assay (17), its EC_50_ in the analogous LRRK2 NanoBRET in-cell target engagement assay was 216 nM, indicating at least 20-fold selectivity for TTK over LRRK2 in the cellular environment (Supplemental Figure 1A; supplemental material available online with this article; https://doi.org/10.1172/jci.insight.177523DS1). These data suggest that TTK is the predominant target of OSU13 in mammalian cells.

TTK is a key regulator of the SAC. Cells without functional TTK can proceed with division even if the spindle assembly is faulty. We have performed a SAC inhibition assay to verify that OSU13 targets the SAC. Nocodazole-arrested CAL-51 human breast adenocarcinoma cells treated with OSU13 or the reference TTKi BOS-172722 showed a dose-dependent decrease in phosphorylated histone H3 (Ser10), indicating the ability of OSU13 to effectively bypass the activated SAC despite the presence of aberrant mitotic spindles (Supplemental Figure 1B).

**Figure 2:**
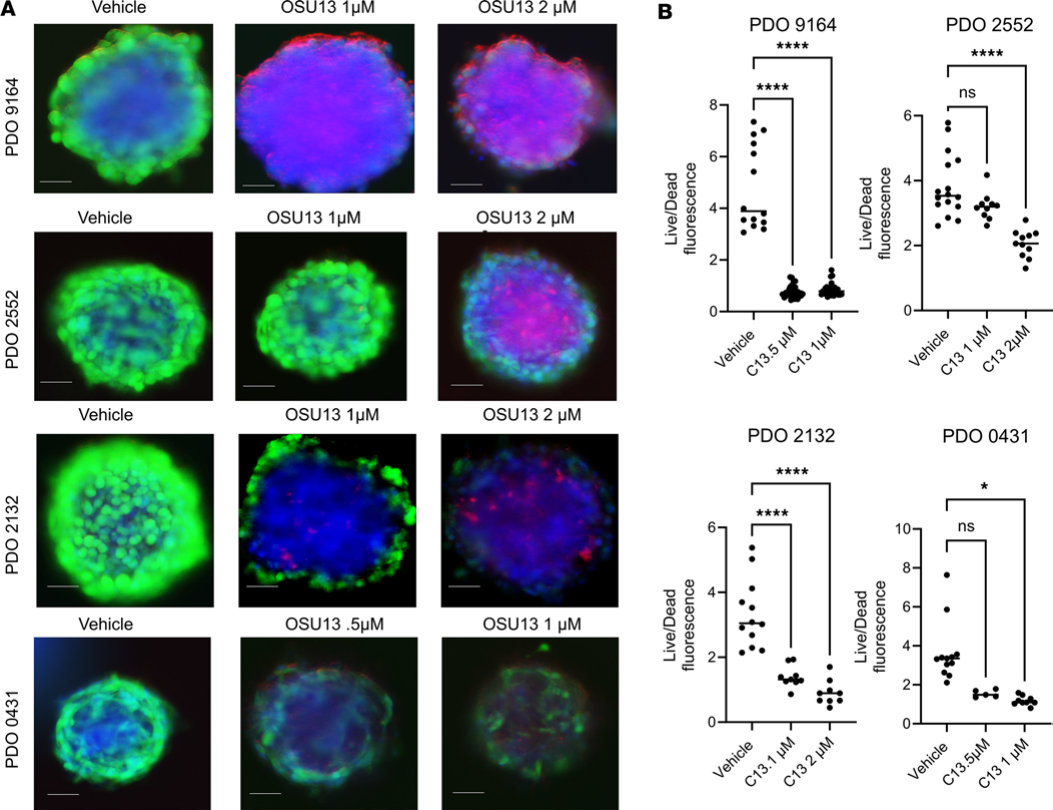
OSU13 reduces organoids derived from patients with melanoma viability. (**A**) Representative images of individual organoids from 4 distinct patients treated with OSU13 at 0.5, 1, or 2 μM for 3 days. Live (green) and dead (red) cells were visualized using Calcein AM and propidium iodide, respectively. DNA (blue) was visualized with Hoechst 33342. Scale bar: 50 μm. (**B**) Quantified data from the PDO experiment shown in **A**. *N* = 8–15 individual organoids per patient and treatment condition. Statistical analysis using ANOVA with Tukey’s multiple comparisons test. *P < 0.05; ****P < 0.0001.

**Figure 3:**
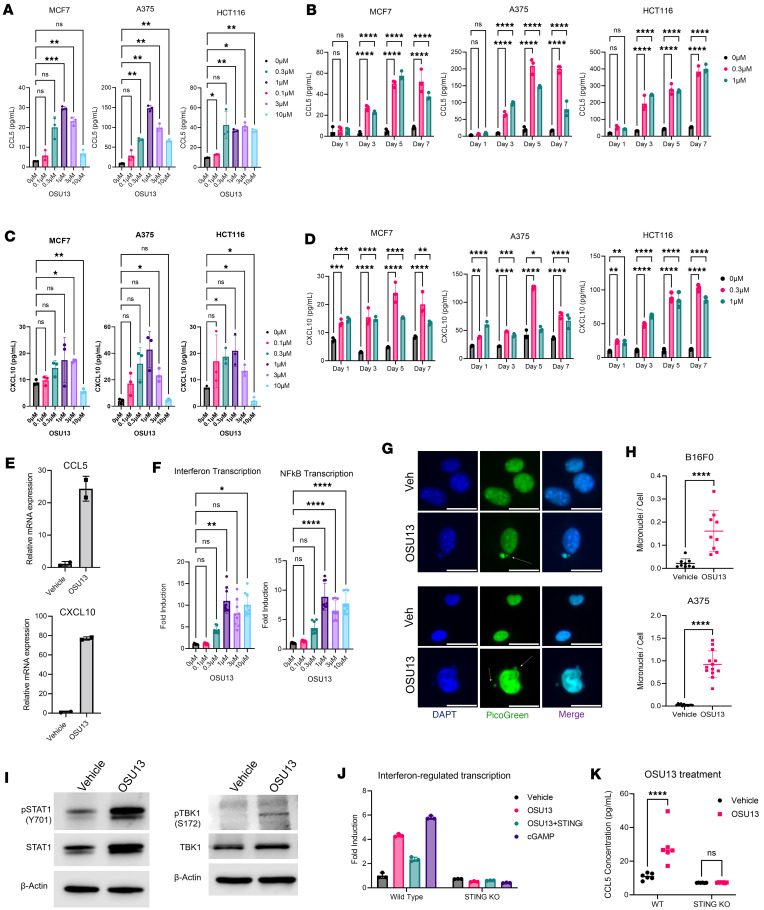
OSU13 induces inflammatory chemokines and transcription factors in a STING-dependent manner. (**A**) Results of ELISA testing for secretion of CCL5 in the conditioned media from cancer cells treated with indicated concentrations of OSU13 for 5 days. Statistical analysis using 1-way ANOVA with Dunnett’s multiple comparisons test. (**B**) CCL5 ELISA results performed on tumor cell–conditioned media collected at 1, 3, 5, and 7 days of treatment with 0.3 or 1 μM OSU13 or vehicle. (**C** and **D**) Same as **A** and **B**, except secretion of CXCL10 was tested. (**E**) Real-time PCR of CCL5 and CXCL10 mRNA expression in MCF7 cells treated with 1 μM OSU13 or vehicle for 5 days. (**F**) Induction of IRF and NF-κB reporters in HCT116 dual cells treated with indicated concentrations of OSU13 for 5 days. *N* = 9 replicates per condition. Statistical analysis using 1-way ANOVA with Dunnett’s multiple comparisons test. (**G**) Representative images of B16F0 (top) and A375 cells (bottom) treated with 1 μM OSU13 or vehicle for 3 days. Micronuclei visualized with pico green and nuclear DNA were labeled with DAPI. White arrows indicate OSU13-treated cells with micronuclei. Vehicle-treated micronuclei^–^ cells are included for comparison. Scale bar: 15 μm. (**H**) Quantification of micronuclei per cell from experiment described in **D**. *N* = 9 microscopic fields per condition. Statistics using *t* test. (**I**) Western blot analysis of STAT1 Y701 phosphorylation and TBK S172 phosphorylation in MCF7 cells treated with 1 μM OSU13 or vehicle for 5 days. (**J**) Induction of IRF/IFN reporter in STING WT and -KO B16-Blue IRF reporter cells treated with 1 μM of OSU13 for 5 days. *N* = 3 replicates. Statistical analysis using ANOVA with Tukey’s multiple comparison test. (**K**) ELISA analysis of CCL5 secretion in STING WT and -KO MC38 cells treated with 1 μM OSU13 for 3 days. *N* = 6 replicates. Statistical analysis using 2-way ANOVA with Šidák’s multiple comparisons test. *P < 0.05; **P < 0.01; ***P < 0.001; ****P < 0.0001.

To test the antitumor effect of OSU13 in a clinically relevant model, we used organoids derived from patients with melanoma previously developed by our group (23, 28). Organoids originating from 4 patients with melanoma were treated for 5 days with OSU13, and live/dead fluorescent viability assay was performed. We detected a statistically significant reduction in cell viability after OSU13 treatment in all tested models (Figure 2, A and B). We next tested potential immunogenic properties of OSU13. We showed above that OSU13-treated cells display features of senescence (Figure 1). In addition to the loss of proliferative potential, senescent cells are known to display SASP, which involves increased secretion of various molecules, including immunoreactive molecules, such as cytokines and chemokines (29). We have previously shown that chemokines CCL5 and CXCL10 play an important role in recruiting immune cells into senescent tumors (19, 21, 22). Thus, we tested if OSU13 promotes CCL5 and CXCL10 secretion by tumor cells using ELISA. We observed a dose- and time-dependent induction of chemokines in all 3 tested cell lines (Figure 3, A–D).

**Figure 4:**
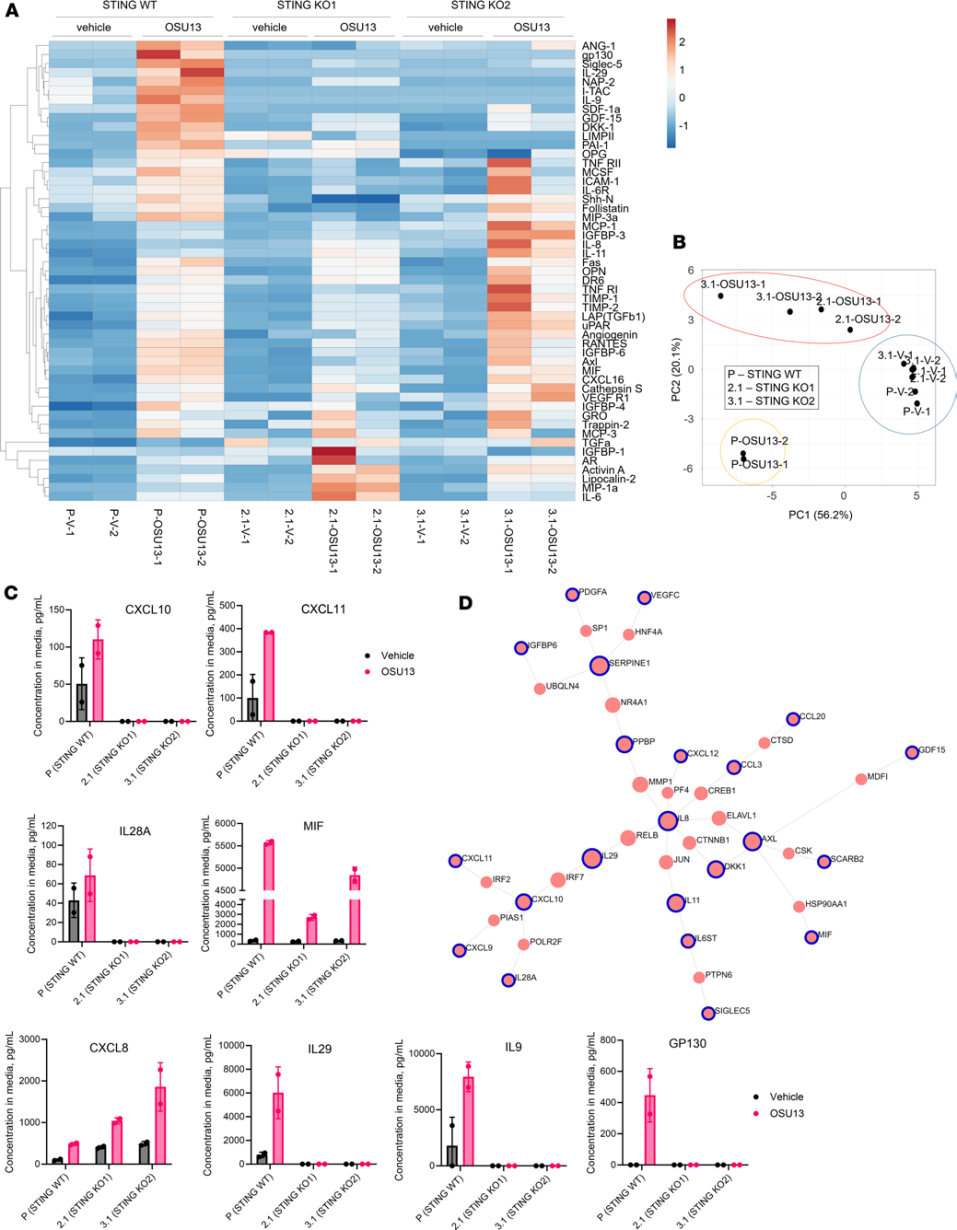
STING promotes a proimmunogenic and tumor-inhibitory secretome in OSU13-treated tumor cells. (**A**) Heatmap depicting relative content of indicated immune proteins in conditioned media from STING WT (p) or -KO (2.1 and 3.1) A375 cells treated for 3 days with vehicle (V) or 1 μM OSU13. Proteins significantly upregulated by OSU13 are shown. Rows are centered; unit variance scaling is applied to rows. Rows are clustered using correlation distance and average linkage. (**B**) Principal component analysis of secreted proteins based on data shown in **A**. (**C**) Secretion of select individual proteins from the data shown in **A**. (**D**) Protein networks constructed based on data in **A**. Circles represent individual proteins differentially expressed in OSU13-treated STING WT and -KO cells. Connecting lines represent known/validated protein-protein interactions. The size of individual circles reflects how many connections this protein has with other network proteins.

Chemokines are commonly regulated on a transcription level. Accordingly, we detected an increase of CCL5 and CXCL10 mRNA in OSU13-treated cells (Figure 3E). We also tested the ability of OSU13 to activate NF-κB and IRF transcription factors that are major regulators of chemokines and inflammatory transcriptional programs. Indeed, the activity of IRF and NF-κB reporters increased in the B16 dual reporter cells after OSU13 treatment (Figure 3F). These innate immune pathways are triggered by pattern-recognition receptors in response to pathogens and danger signals. Based on the knowledge that TTK is important for chromosome segregation, we postulated that OSU13 might trigger inflammatory transcription by inducing micronuclei, which are extranuclear DNA-containing structures that form by unsegregated chromosome fragments or lagging chromosomes. As predicted, we observed an increased proportion of cells with micronuclei after OSU13 treatment (Figure 3, G and H). Micronuclei and DNA damage are reported to activate the cyclic GMP–AMP synthase/stimulator of IFN genes (cGAS/STING) pathway, which is a pattern-recognition receptor pathway responsible for detecting and responding to cytosolic DNA (30, 31). Notably, OSU13 induced micronuclei and DNA damage in cancer cells (Figure 3, G and H, and Figure 1, C and D). Accordingly, we detected increased phosphorylation of downstream STING pathway nodes, such as STAT1 and TBK1, in OSU13-treated cells (Figure 3I). In addition, the induction of IRF reporter was diminished in the presence of STING inhibitor (Figure 3J). Furthermore, the induction of IRF reporter and CCL5 secretion by OSU13 was abrogated in mouse melanoma and human colon cancer cells with *Sting* gene KO (Figure 3, J and K). To confirm that STING is responsible for the OSU13-induced inflammatory phenotype in human cancer cells, we generated 2 A375 human melanoma cell lines with STING KO using CRISPR/Cas9 editing (Supplemental Figure 2). Multiplex ELISA analysis revealed that STING-KO cells displayed a distinct secretome compared with parental STING WT cells (Figure 4A). This was further validated by a principal component analysis, in which OSU13-treated STING-KO cells were stratified from OSU13-treated WT cells on an PCA plot (Figure 4B). To gain further insight into the role of STING in regulation of OSU13-triggered inflammatory phenotype, we selected proteins differentially secreted by OSU13-treated STING WT and STING-KO cells for further analysis (Figure 4C). Only proteins that were up- or downmodulated in both STING-KO clones with *P* values of less than 0.1 were chosen (Supplemental Table 1). The induction of T cell–recruiting chemokines CXCL10 and CXCL11 was abrogated by STING KO. Similarly, cytokines known to promote cytotoxic T cell responses, such as type III interferons IL-28A and IL-29, were not induced in the absence of STING. In addition, loss of STING also limited the OSU13-mediated induction of MIF that suppresses antiinflammatory effects of glucocorticoids, which play pivotal role in tumor immune evasion (32). Another intriguing effect of STING KO was a complete loss of the treatment-induced IL-9, which has been implicated in antitumor immunity and immunotherapy responses (33–36).

Interestingly, there were a number of proteins that displayed increased secretion in OSU13-treated cells with STING KO compared with STING WT cells, suggesting that STING signaling downmodulates their induction in response to stress. One such protein is CXCL8 (IL-8), a chemotactic cytokine for granulocytic cells implicated in tumor promotion (Figure 4C). We also observed a decrease of the secretion of GP130 (IL6ST) in STING-KO cells. Soluble GP130 inhibited signaling from IL-6 and other tumor-promoting cytokines (Figure 4C). Of note, secretion of IL-6 and IL-8 is a key characteristic of senescent cells and an integral component of SASP linked to chronic inflammation and tumor promotion (37). Thus, the observed modulation of IL-8 and IL-6 signaling by STING loss suggests that STING activation may limit tumor-promoting effects of senescence and SASP.

Using a knowledgebase of reported and predicted protein-protein interactions, we constructed a network connecting proteins differentially secreted by OSU13-treated WT and STING-KO cells (Figure 4D). A list of proteins used as input is provided in Supplemental Table 2. IL-8 (CXCL8) was identified as the most interconnected protein within the network, suggesting its potential importance in STING-mediated regulation of the phenotype of OSU13 response. Altogether, the network analysis suggested that STING regulates the secretory phenotype of tumor cells in response to OSU13 to enhance antitumor immunity and limit tumor-promoting inflammation.

**Figure 5:**
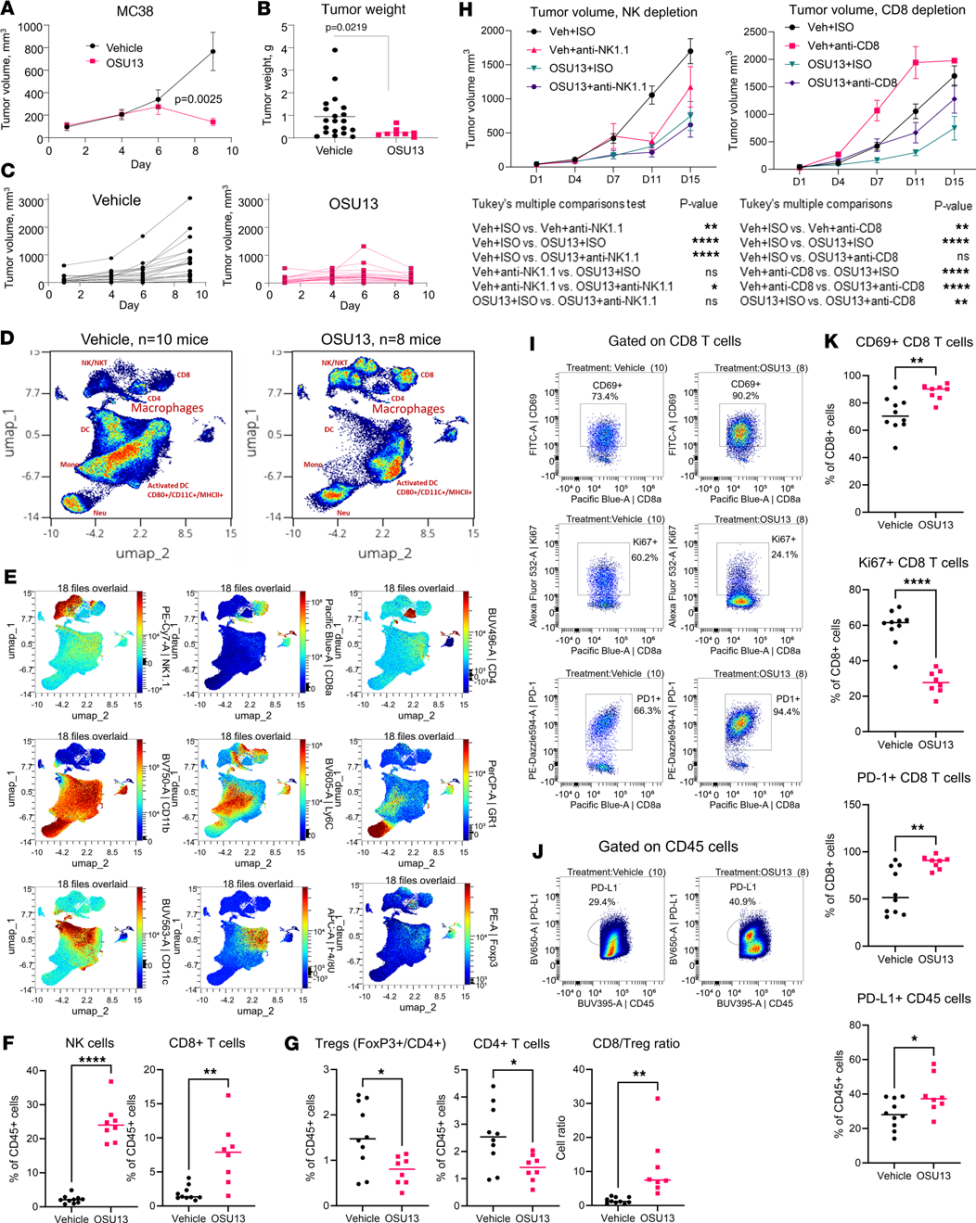
OSU13 inhibits tumor growth and increases tumor immune infiltrate. (**A**) Growth of MC38 tumors in mice treated with 10 mg/kg OSU13 or vehicle 5 days a week (5 days on/2 days off). Statistical analysis using mixed model. *N* = 20. (**B**) Final weight of the tumors from the experiment shown in **A**. (**C**) Growth of individual tumors from the experiment shown in **A**. (**D**) Dimension reduction analysis of the spectral cytometry data from tumors shown in **A**. Only CD45^+^ cells were used for UMAP construction. Red labels indicate the suggested identity of select cell populations based on marker expression shown in **E**. (**E**) Expression of indicated immune markers on CD45^+^ cells from tumors in **A**. (**F** and **G**) Percentages of NK1.1^+^ cells, CD8^+^ T cells, and FoxP3^+^/CD4^+^ Tregs in total CD45^+^ tumor infiltrate based on manual gating. (**H**) Tumor growth in NK cell–depleted (anti-NK1.1), CD8^+^ T cell–depleted (anti-CD8), or nondepleted (ISO) mice treated with vehicle or 10 mg/kg OSU13 5 days a week. *N* = 12–13 mice per group. Statistics using mixed model. (**I**) Expression and gating of indicated phenotype markers in CD8^+^ T cells from tumors shown in **A**. (**J**) Expression and gating of PD-L1 in CD45^+^ cells from tumors shown in **A**. (**K**) Summary of spectral cytometry data showing expression of indicated phenotype markers on CD8^+^ T cells and total CD45^+^ cells from tumors in **A**. *P < 0.05; **P < 0.01; ****P < 0.0001.

To determine if OSU13 has antitumor and proimmunogenic activity in vivo, we established subcutaneous tumors in immunocompetent C57BL/6 mice using MC38 murine colon carcinoma cells. Treatment was administered daily without a break for 9 days by oral gavage. We noted diarrhea developing after a week of dosing in the OSU13 group, and several mice in this group died before the experiment’s completion. We adjusted the schedule to 5 days on and 2 days off in all of the following experiments to minimize toxicity. There was a prominent antitumor effect with the continuous dosing of OSU13, with statistically significant tumor growth inhibition (Figure 5, A and C) and reduction in the final tumor weight (Figure 5B). Spectral cytometry analysis revealed an enrichment of NK and CD8^+^ T cells and a reduction of Tregs within the total CD45^+^ tumor immune infiltrate in the OSU13-treated mice (Figure 5, D and E). This conclusion was validated using unbiased clustering analysis of the spectral cytometry data that showed enrichment of clusters of cells expressing NK and CD8^+^ T cell markers in tumors from OSU13-treated mice (Supplemental Figure 3, A and B). Traditional gating based on NK1.1^+^ and CD8^+^ surface markers also confirmed an increase in the proportion of NK and CD8^+^ T cells infiltrating tumors in OSU13-treated mice (Figure 5F). On the contrary, the proportion of Foxp3^+^CD4^+^ cells (Tregs) was significantly reduced in mice treated with OSU13, resulting in an overall reduction in CD4^+^ T cells (Figure 5G). On further analysis we found that OSU13 treatment significantly enhanced CD8-to-Treg ratio (Figure 5G).

To determine whether NK cells or CD8^+^ T cells recruited by OSU13 treatment contributed to antitumor activity of this therapeutic, we used the NK cell and CD8^+^ T cell depletion approach (Supplemental Figure 4, A–D). Tumor inhibition by OSU13 was not dramatically affected by NK cell depletion. In contrast, OSU13-treated tumors grew faster in mice depleted of CD8^+^ T cells compared with nondepleted controls (Figure 5H). Based on these findings, we further investigated the phenotype of CD8^+^ T cells in OSU13-treated tumors. A greater proportion of tumor-infiltrating CD8^+^ T cells expressed the activation marker CD69 after OSU13 treatment compared with no-drug control. However, CD8^+^ T cells also displayed higher expression of exhaustion marker PD-1 and lower expression of proliferation marker Ki67^+^ after OSU13 treatment (Figure 5I). The expression of CD69 and PD-1 on CD4^+^ T cells was not significantly affected by OSU13, while the proportion of Ki67^+^CD4^+^ T cells was reduced (Supplemental Figure 4, E and F). These data suggest that CD8^+^ T cells were engaged in antitumor activity during OSU13 treatment; however, their activity was restrained by PD-1 immune checkpoint. Furthermore, the increase of PD-L1–expressing CD45^+^ cells in tumors of OSU13-treated mice also suggests the engagement of PD-1/PD-L1 immune checkpoint (Figure 5J). Quantification and statistical analysis of the phenotype markers discussed above is shown on Figure 5K.

**Figure 6:**
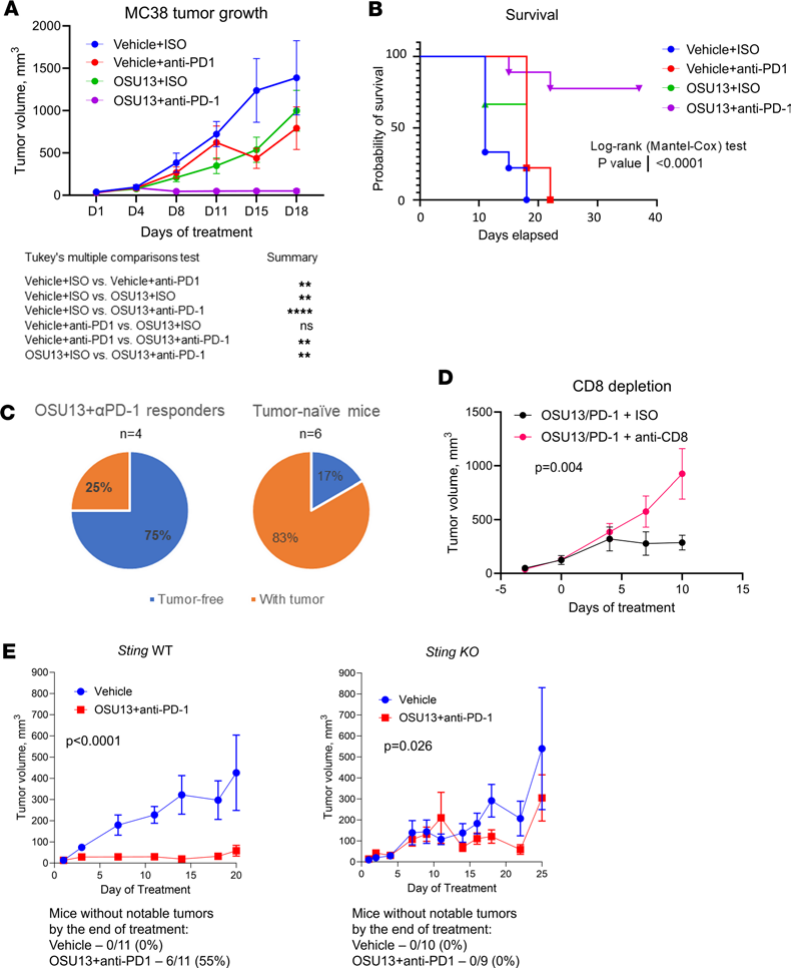
OSU13 augments immunotherapy responses in mice. Growth of MC38 tumors treated with vehicle or OSU13 (10 mg/kg, 5 days a week) in the absence or presence of 100 μg anti–PD-1 or isotype antibody treatment (dose). *N* = 6–10 mice per group. Statistics using a mixed model with Tukey’s post test. (**B**) Survival of mice shown in **A**. Statistical comparison between vehicle and OSU13 and anti–PD-1 combination group is indicated. (**C**) Results of the tumor rechallenge experiment. MC38 tumor cells were injected in 4 mice that rejected tumors after OSU13 and anti–PD-1–treatment (from experiment in **A**) and in 6 tumor-naive mice. Rechallenge was performed 2 months after therapy completion. Areas in blue indicate mice that did not develop tumors. (**D**) MC38 tumor growth in CD8^+^ T cell–depleted and nondepleted mice treated with OSU13 and anti–PD-1. *N* = 8 mice per group. Statistics using mixed model. (**E**) Response of WT and STING-KO tumors to OSU13 and anti–PD-1 combination treatment. *N* = 9–11 mice per group. Statistics using mixed model. **P < 0.01; ****P < 0.0001.

To overcome the PD-1/PD-L1 immune checkpoint, we treated mice bearing MC38 tumors with OSU13 and checkpoint-blocking anti–PD-1 antibody. MC38 tumors are commonly used for studies of ICB and have been shown to exhibit low-to-moderate response to anti–PD-1 that is CD8^+^ T cell dependent (38). There was partial inhibition of tumor growth with anti–PD-1 alone and OSU13 alone. Notably, when both treatments were combined, there was complete inhibition of growth (Figure 6A). Furthermore, mice treated with the OSU13 and PD-1 combination displayed improved survival compared with other treatment groups (Figure 6B). Mice that achieved complete response on OSU13 and anti–PD-1 therapy and remained tumor-free for 2 months after treatment were rechallenged with the same tumor. Only 1 of 4 mice (25%) developed a tumor after a rechallenge. This contrasts with the 83% of mice that developed tumors in the tumor-naive group (Figure 6C). This suggests a presence of immunologic memory against tumor antigens in mice that responded to OSU13 and PD-1 combination therapy. To confirm that the antitumor immune response in OSU13- and anti–PD-1–treated mice was CD8 T cell–dependent, as we previously observed with OSU13 alone (Figure 5H), we compared MC38 tumor inhibition in CD8^+^ T cell–depleted and nondepleted mice. The inhibitory effect of OSU13 and anti–PD-1 therapy was abrogated in CD8^+^ T cell–depleted mice (Figure 6D). We also tested the OSU13 and anti–PD-1 combination in an alternative model in which murine CT26 colon carcinoma tumors were grown in BALB/c mice. In this model, a combination of a 50 mg/kg OSU13 administered once a week with anti–PD-1 immunotherapy demonstrated statistically significant inhibition of tumor growth compared with vehicle and single-agent treatments and improved survival in mice (Supplemental Figure 5, A and B).

Our in vitro data showed that the OSU13-mediated induction of interferon responses, chemokine CCL5, and several other proimmunogenic factors in tumor cells was STING dependent. Therefore, we next asked if the stimulation of antitumor immunity by OSU13 and anti–PD-1 therapy is dependent on STING activation in tumor cells. We injected mice with WT or STING-KO tumor cells and treated them with OSU13 and anti–PD-1. As expected, treatment abrogated the growth of STING WT tumors (Figure 6E). Of 11 mice in the treatment group, 6 showed tumor regression to the point at which they became unnoticeable/unmeasurable (1 mm or less in diameter). We also observed statistically significant growth inhibition in mice inoculated with STING-KO tumors. However, the antitumor effect was less potent. Furthermore, none of the treated mice showed prominent tumor regression (Figure 6E). The partial growth inhibition of STING-KO tumors could be explained by the direct inhibitory effect of OSU13 on tumor cells. These data suggest that activation of tumor cell-intrinsic STING is a key mechanism of antitumor immunity stimulation by OSU13.

**Figure 7:**
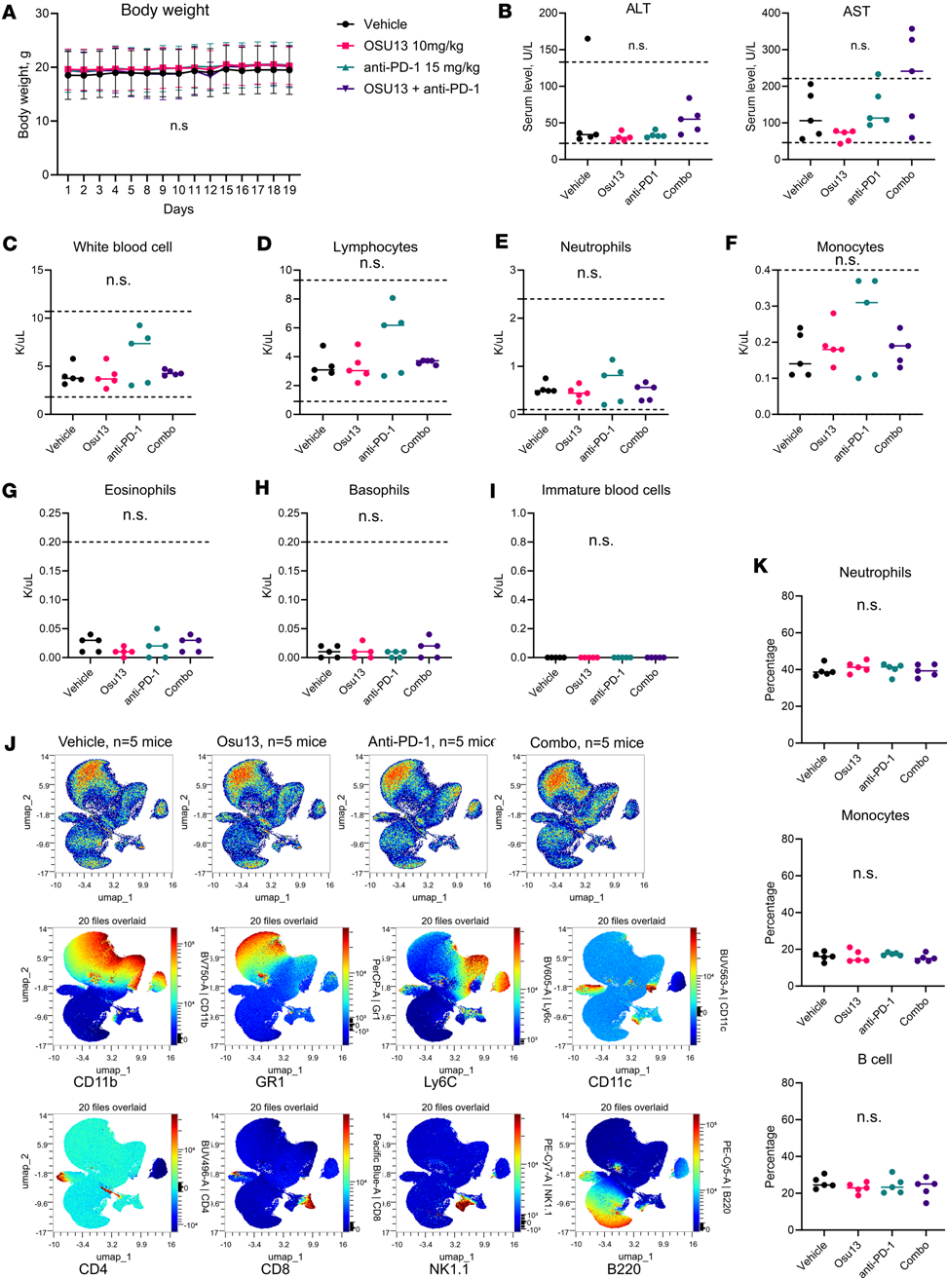
OSU13-treated C57BL/6 mice display no signs of severe toxicities when treated 5 days a week. (**A**) Changes in body weight over time in tumor-free C57BL/6 mice. Mice were treated with OSU13 5 days a week or anti–PD-1 twice a week for 3 weeks. *N* = 5 mice per group. (**B**) Analysis of indicated liver proteins in the serum of mice described in **A**. The dotted lines indicate the normal range for C57BL/6 mice. (**C**–**I**) Analysis of white blood cells in mice whole blood. The dotted lines indicate the normal range for each type of cell. (**J**) Spectral cytometry analysis of bone marrow cells from mice described in **A**. Second and third row indicate the dimension reduction analysis and distribution of select immune marker expression. (**K**) Percentages of indicated cell types within the total live bone marrow cells from the analysis described in **J**. Viable cells were gated based on positivity for GR1, Ly6C, and B220 markers and analyzed using 1-way ANOVA.

Finally, we investigated potential side effects that may occur with OSU13 treatment. Tumor-free C57BL/6 (Figure 7) and BALB/c (Supplemental Figure 6) mice were treated with 10 mg/kg OSU13 or drug vehicle for 3 weeks on a 5 days on/2 days off schedule. C57BL/6 mice were also treated with anti–PD-1 or an anti–PD-1 and OSU13 combination to test for potential unexpected toxicities from combining OSU13 with immunotherapy. There were no significant changes in body weight, indicating no severe GI toxicities with the tested dosing schedule (Figure 7A and Supplemental Figure 6A). Serum levels of liver enzyme alanine aminotransferase (ALT) were not significantly affected in any of the treatment groups and were within the normal range in C57BL/6 and BALB/c mice. The levels of aspartate aminotransferase (AST) were slightly elevated in the C57BL/6 mice; however, this increase was not statistically significant (Figure 7B). The AST serum levels did not exceed the normal range in either of the groups in BALB/c mice (Supplemental Figure 6B). We also performed a complete blood count in C57BL/6 mice to test for potential cytopenia. No changes to total white blood cells, lymphocytes, neutrophils, monocytes, eosinophils, basophils, and immature blood cells were detected in any of the treatment groups, and all values were within the normal range (Figure 7, C–I). Finally, we tested for potential bone marrow toxicities (myelotoxicities). Spectral cytometry analysis of the bone marrow from C57BL/6 and BALB/c mice revealed no major myelotoxicities, as the relative distribution of different myeloid cell populations was comparable in the vehicle group and any of the experimental treatment groups (Figure 7J and Supplemental Figure 6, C and D). Traditional gating also did not reveal statistically significant changes in the neutrophil, monocyte, or B cell populations within the bone marrow (Figure 7K).

In summary, we show that OSU13 exhibited antitumor activity against various tumor types, stimulated CD8^+^ T cell–mediated antitumor immune responses, and improved responses to ICB therapy.

## Discussion

Here, we investigated the mechanism of antitumor activity of a TTKi, OSU13, to identify promising combination strategies for clinical development of this drug and its derivatives. Our results indicated a strong rationale for combining OSU13 with ICB. First, we found that OSU13-treated tumor cells activated a proimmunogenic transcriptional program that translated to increased secretion of chemokines CCL5 and CXCL10, which are known to recruit activated T cells and other immune effectors to the sites of inflammation (39). Accordingly, mice treated with OSU13 displayed enrichment of immune effectors of the adaptive and innate immune system, such as CD8^+^ T cells and NK cells, within the immune infiltrate of their tumors. Lack of immune infiltrate into the tumor microenvironment, specifically T cells, has been linked with poor patient prognosis and low likelihood of responding to ICB (40). Furthermore, it has been suggested that recruitment of T cells into the tumor during ICB is an important determinant of therapeutic outcome (41).

Second, OSU13-treated cells secreted proteins known to facilitate antitumor immunity, such as type III interferons IL-28A and IL-29. These cytokines display type I interferon-like antiviral and antitumor activities (42, 43). Furthermore, OSU13 induced secretion of soluble gp130 from tumor cells. Gp130 is most known for its function as a transmembrane signal-transducing receptor that forms part of the receptor complex for several cytokines, including IL-6 (44). However, in its soluble secreted form, gp130 acts as an inhibitor of IL-6 signaling (45, 46). IL-6 signaling drives the proliferation, survival, invasiveness, and metastasis of tumor cells, while strongly suppressing the antitumor immune response (47). Thus, secretion of an inhibitor of IL-6 signaling by OSU13-treated cells is likely to limit tumor progression and promote antitumor immunity. In addition, OSU13-treated tumor cells produced IL-9. IL-9 garnered increased attention based on a number of recent studies that demonstrated its powerful antitumor role in solid tumors. It is reported to have a direct inhibitory effect on tumor cell proliferation and metastasis and a tumor-extrinsic activity via promotion of innate and adaptive antitumor immunity and immunotherapy response (33–36). Conventionally, production of IL-9 is attributed to Th9 cells. Thus, the fact that tumor cells themselves can secrete IL-9 in response to drugs, such as OSU13, is intriguing and warrants further investigation beyond this study.

Of note, increased secretion is a key characteristic of cellular senescence, which is a type of stress response associated with damage and loss of proliferative potential that often occurs in tumors undergoing therapy. SASP is extremely diverse and can have pro- and antitumor effects depending on the damaging stimuli and type of cells undergoing senescence. The cytosolic DNA sensor STING has been implicated as an inducer of SASP (48, 49). Contrary to this notion, we observed increased secretion of immune-related proteins in both STING WT and -KO cells after OSU13 treatment. However, loss of STING completely abrogated secretion of the proteins with antitumor activity mentioned above, such as CXCL10, CXCL11, IL-28A, IL-29, IL-9, and GP130. Thus, it is plausible that, in the context of OSU13 treatment, STING acted not as the inducer of SASP, but rather a SASP modifier that established a secretome favorable to tumor growth inhibition, tumor surveillance by T cells, and antitumor immune response. Biologically, the link between STING activation and cytotoxic immune responses makes sense. STING is a key factor of immune defense against DNA viruses (50). When viral DNA is present inside the infected cells, activation of STING would arrest those cells and promote their T cell–mediated killing. In contrast, after the virus is cleared and the STING pathway is no longer triggered, it would be more beneficial to secrete factors that inhibit cytotoxic T cell responses and facilitate proliferation to promote tissue repair.

The third observation in support of combining OSU13 with immunotherapy came from analysis of tumor immune infiltrate, which revealed increased expression of PD-1 on T cells from OSU13-treated mice. We also observed that CD45^+^ cells from OSU13-treated mice expressed higher levels of PD-L1 on their surface. These phenotypic changes are indicative of activation of the PD-1–PD-L1 immune checkpoint that likely limited the amplitude of antitumor immune response induced by OSU13 treatment. Based on these 3 factors, we tested OSU13 in combination with anti–PD-1 antibody and observed prominent T cell–dependent tumor inhibition and improved survival in mice. Notably, a study by another group reported improved response to PD-1 blockade in mice treated with a different TTKi, CFI-402257, suggesting that the induction of proimmunogenic phenotype by OSU13 is likely an on-target effect of TTK inhibition and, therefore, other TTKi are likely to compliment immunotherapy as well (6).

Our studies into the mechanism of proimmunogenic activity of OSU13 revealed the essential role of the antiviral cGAS/STING pathway. Recently, it has been appreciated that the STING pathway can be induced by nonviral stimuli. For example, the presence of micronuclei, small DNA compartments encapsulated by a nuclear envelope and spatially separated from the primary nucleus, has been linked with STING activation. Rupture of micronuclei causes the release of the DNA content into the cytosol, where it is recognized by the STING activator cGAS (30). Furthermore, severe DNA damage has been reported to activate STING presumably due to the escape of broken DNA fragments from the nucleus (31). We showed that OSU13 induced micronuclei and DNA damage in tumor cells. This is likely to be an on-target effect, based on prior studies of other TTKi reporting micronuclei and STING activation (5, 51). Interestingly, one study reported that TTK loss can cause chromatin bridges that also activate cGAS/STING pathway (52). Notably, we showed that genetic ablation of STING in tumor cells desensitized mice to OSU13 and anti–PD-1 therapy. These data point to an essential role of the tumor-intrinsic STING in establishing effective antitumor immunity.

According to pan-cancer analysis, a subset of tumors (up to 25%) downregulates the STING pathway through epigenetic silencing of genes encoding STING or cGAS (53). While the exact biological outcome of STING loss in tumors is not fully understood, it has been suggested as a potential mechanism of tumor immune escape (53–55). Notably, a recent study showed that KRAS-LKB1 mutant lung cancer that often silences the STING pathway is resistant to TTKi and immunotherapy combinations; however, epigenetic drugs can improve response by inducing STING reexpression (51). Considering the dichotomy of STING pathway silencing in tumors, the status of STING and cGAS could be used as biomarkers for patient recruitment onto an OSU13 and anti–PD-1 clinical trial. An effective biomarker would improve the response rates, prevent unnecessary treatment for trial participants, and increase the overall likelihood of establishing TTKi as an effective tool for boosting immunotherapy efficacy in clinic. This is an unmet clinical need, as no reliable TTKi response biomarkers have been developed to date. In fact, a study of TTKi CFI-402257 revealed no correlation between treatment response and key cancer-associated mutations (e.g., in APC, BRAF, CDKN2A, PIK3CA, RAS, and TP53) or cell doubling time across a panel of cancer cell lines (6). Of note, a recent clinical study revealed that STING expression is a biomarker for overall survival in PDL1^–^, TMB^lo^ non–small cell lung cancer treated with ICB (56). This further supports the idea to use STING as a marker for recruiting patients on TTKi and ICB trials.

One caveat of our study is that we focused on one TTKi, OSU13, which means we cannot fully exclude the possibility that some of the observed effects could be off target. However, the kinase activity and SAC inhibition assay data presented here and previously (18) indicate that TTK is a predominant target of OSU13 in cells. Based on our findings, we foresee the use of TTKi for pharmacological engineering of antitumor immunity in tumors with a functional STING pathway. For example, TTKi can be used to jump start the immune response against tumors that are poorly immunogenic, such as tumors with low mutational burden and minimal T cell infiltrate. Because tumors with the evidence of ongoing immune response are more likely to respond to ICB immunotherapy compared with “immune-cold” tumors, further exploration of OSU13 and other TTKi in combination with ICB clinically could be promising.

## Methods

### Sex as a biological variable.

Our study examined male and female animals, and similar findings are reported for both sexes.

### Cell lines and PDOs.

A375, HCT116, MCF7, MC38, and CT26 cells were purchased from ATCC. B16-IRF reporter and HCT116 dual reporter cells were purchased from Invivogen. MC38-STING-KO cells were gifted by Anli Zhang at UT Southwestern (Dallas, Texas, USA). A375-STING-KO cells were generated from A375-Cas9 (ABM) cells by transfecting them with a plasmid encoding sgRNA targeting STING (ABM, catalog 469411110191). Cells were cultured in Dulbecco’s modified Eagle’s medium/F12 medium supplemented with 10% FBS and 1% penicillin-streptomycin (Gibco). Patient-derived organoids (PDOs) were generated and described previously (23). Organoids were cultured in complete media containing DMEM-F12 (Gibco), 15% FBS (Gibco), 1X B27-supplement (Gibco), and 5% Matrigel (Corning) in ultra-low attachment plates.

### Cell viability, senescence-associated β-Gal staining, and micronuclei assay.

Cells were seeded at 0.2 × 10^6^ to 0.3 × 10^6^ cells per well in flat-bottom 6-well plates and allowed to attach overnight prior to treatment and treated as described in figure legends. Cells were photographed in phase-contrast using an EVOS microscope and counted by a pathologist. For crystal violet staining, cells were fixed with 100% methanol (MilliporeSigma) for 10 minutes and stained with 1% (w/v) crystal violet (MilliporeSigma) for 15 minutes, followed by at least 3 washes with water.

The fluorescent viability assay in organoids was described previously (23). Briefly, PDOs were seeded in an ultra-low attachment 96-well plate. OSU13 or vehicle (DMSO) was added to the wells in triplicate, and PDOs were incubated for 72 hours. To visualize dead cells, DNA, and live cells, cultures were incubated with 50 μg/mL propidium iodide, 10 μg/mL Hoechst 33342, and 5 μM Calcein AM (Thermo Fisher Scientific), respectively. Images were taken on an inverted fluorescent microscope (Thermo Fisher Scientific, EVOS M7000). Live (green) and dead (red) cells per organoid region were quantified by ImageJ (NIH). Organoid regions were identified in a trans channel image. The ratio of live to green cell counts were plotted.

Senescence-associated β-galactosidase was performed using a kit (Senescence Cells Histochemical Staining Kit, catalog CS0030, MilliporeSigma) according to the manufacturer’s protocol. Micronuclei were detected using PicoGreen (Quant-iT PicoGreen dsDNA Reagent, Thermo Fisher Scientific). Cells were incubated for 1.5 hours with 3 μL per mL of PicoGreen diluted in complete medium and subsequently fixed in 4% paraformaldehyde in PBS. Coverslips were mounted with ProLong Diamond Antifade Mountant (Invitrogen). Images were acquired from 4 representative fields of each condition using an inverted fluorescent microscope (EVOS M7000, Thermo Fisher Scientific). The number of micronuclei per cell was quantified in ImageJ using the Cell Counter plugin. Micronuclei structures were defined as minor DNA aggregates separate from the primary nucleus. In addition to PicoGreen staining, DAPI and transmitted light images were used to confirm micronuclei structure and localization.

### Western blot.

Cells were lysed with RIPA buffer (MilliporeSigma) supplemented with protease inhibitors (MilliporeSigma). Protein concentrations were determined using Bradford Protein Assay reagent (Bio-Rad) according to the manufacturer’s protocol. Primary antibodies detecting cyclin A2 (91500S), p21 (2947S), phospho-STAT1 (9167S), STAT1 (14994S), phospho-TBK1 (5483T), and TBK1 (3013S) were purchased from Cell Signaling. Primary antibodies were hybridized overnight at 4°C at dilutions of 1:500, except for housekeeping proteins β-actin (4970S), β-tubulin (2146S), and HSP90 (4874S) (all from Cell Signaling), which were used at 1:2,000. Secondary antibodies (Cell Signaling) were used at a dilutions of 1:10,000 for 2 hours at room temperature.

### Real-time PCR, ELISA, multiplex ELISA, and bioinformatics analysis.

CCL5 mRNA was measured using real-time PCR as described previously (19). Human CCL5 and CXCL10 secretion was assessed using ELISA kits purchased from R&D Systems. Mouse CCL5 was detected using an ELISA kit from R&D Systems. For the analysis of cell secretome, cell-conditioned media was submitted to RayBiotech for quantitative multiplex ELISA assay that provided absolute concentrations of tested proteins in the media. These data were used to calculate PCA, plot the heatmap, and identify proteins differentially expressed between treatment group. To construct a PCA plot and heatmap, expression data were uploaded to ClustVis web portal. Individual proteins were organized in rows, and treatment conditions in columns. For PCA, unit variance scaling was applied to rows; SVD with imputation was used to calculate principal components. The *x* and *y* axes show principal component 1 and principal component 2. For heatmaps, rows were centered; unit variance scaling was applied to rows. Both rows and columns were clustered using correlation distance and average linkage.

To identify proteins differentially expressed between OSU13-treated STING WT and -KO cells multiple unpaired *t* test was used in GraphPad Prism with individual variances computed for each protein. Proteins were considered to be differentially expressed if the *t* test *P* value was smaller than 0.1. A list of proteins that passed the FDR threshold was uploaded to OmicsNet, and network analysis of one list of molecules was performed using the “Proteins” option. STRING database of known and predicted protein-protein interactions was selected for network creation and Streiner Forest (PCSF) network tool was applied to simplify the network by identifying subnetwork enriched with input values. The networks were visualized in 2D.

### Mouse experiments.

The experiment with CT26 tumors in BALB/c mice was performed by Charles River Laboratories. All other animal experiments were approved by The Ohio State University IACUC. Male and female C57BL/6 mice purchased from The Jackson Laboratory were used for in vivo tumor experiments. C57BL/6 and BALB/c mice were used for toxicity studies. To inoculate CT26 tumors, female mice were injected subcutaneously with 3 × 10^5^ cells. To inoculate MC38 tumors, 10^5^ cells were injected subcutaneously into male and female mice. Tumors were measured using calipers, and tumor volume was calculated as 0.5 × length × width × width. Mice were euthanized if their tumors exceeded 16 mm in diameter or became perforated or if a mouse lost more than 20% body weight.

OSU13 was provided by The Ohio State University Comprehensive Cancer Center Drug Development Institute. The vehicle used to prepare dosing solution of OSU13 was 12.5% ethanol/12.5% Cremophor EL (MilliporeSigma) in 5% dextrose in water. OSU13 was prepared by dissolving an appropriate amount of powder stepwise in ethanol, Cremophor EL, and D5W to yield dosing solutions at 1.16 and 5.78 mg/mL in vehicle. These provided active doses of 10 and 50 mg/kg, respectively.

For CT26 experiments, anti–PD-1 RMP1-14 (Ichor, lot IB3207) and isotype control rat IgG2a clone 2A3 (BioXCell, lot 749620N1) were used at 5 mg/kg by i.p. injection twice a week. For MC38 experiments, anti–PD-1 clone 29F.1A12 (BioXcell) or control Rat IgG2a clone 2A3 (BioXcell) were used at 100 μg by i.p. injection twice a week. For the in vivo cell depletion experiment, anti-CD8 (clone 53-5.8) and anti-NK1.1 (clone PK136), both from BioXCell, were injected i.p. at 100 μg per mouse every 3 days.

For the studies of toxicities, terminal blood collection was performed by severing the brachial plexus with scissors. For white blood cell count, blood was collected into EDTA-containing BD vacutainer tubes (catalog 367861); for AST and ALT analysis, blood was collected in 1.5 mL microcentrifuge tubes, allowed to clot, and centrifuged to obtain the serum. Samples were submitted to the Comparative Pathology and Digital Imaging Shared Resource (also known as CPDISR, part of the The Ohio State University Comprehensive Cancer Center) for the following services: hematology (complete blood count/differential) and single chemistry (AST and ALT).

### Flow cytometry.

Spectral flow cytometry was used to study the expression of surface or intracellular (nuclear) immune markers (Supplemental Table 3) in bone marrow and tumor-derived immune cells. Mouse bone marrow cells were obtained by flushing mouse tibias and femurs with PBS. To obtain tumor cell suspensions, tumors were minced and processed on a gentleMACS dissociator in the presence of tissue digesting enzymes (Miltenyi Biotech). Cell suspensions were filtered through a cell strainer (BD) and incubated with FC blocking antibody (BD Pharmingen) and a fixable viability dye (Live/Dead Aqua, Thermo Fisher Scientific). After a 30-minute incubation with fluorescent antibodies recognizing surface markers, FOXP3 staining was performed using the FOXP3/Transcription factor staining buffer set (eBiosciences/Invitrogen, 00-5523-00) according to the manufacturer’s instructions. Cells were fixed in 0.5% buffered paraformaldehyde and analyzed with a 5-laser Cytek Aurora. We used the UMAP algorithm implemented in OMIQ software for dimensionality reduction of data. FlowSOM clustering algorithm with consensus clustering metaclustering was used to identify distinct cell populations. Immune cell clusters were annotated by visually investigating the expression of immune markers in cells distributed on UMAP space.

### NanoBRET in-cell target engagement assay.

HEK293 cells were transiently transfected with 1 μg LRRK2-NanoLuc fusion vector and 9 μg transfection carrier DNA. The transfected cells were treated for 1 hour with several concentrations of OSU13 (3-fold dilutions starting at 5 μM) or the positive control receptor protein tyrosine kinase inhibitor CEP-701 (3-fold dilutions starting at 1 μM). Kinase-ligand affinity was measured by competitive displacement with 0.016 μM K-9 tracer. The 600 nm/460 nm ratio was calculated, and the normalized BRET response (percentage) was established prior to curve fitting.

### SAC inhibition assay.

CAL-51 cells were incubated with 100 ng/mL nocodazole for 20 hours and subsequently treated with several concentrations of OSU13 or the reference TTKi BOS-172722 (3-fold dilutions starting at 1 μM) without removal of nocodazole. After 2 hours, the cells were washed once with cold PBS, lysates were prepared, and phospho-histone H3 (Ser10) was measured using the Meso Scale Discovery Phospho-Histone H3 (Ser10) Assay Whole Cell Lysate Kit (catalog K150EWD-2).

### Statistics.

Two-tailed Student’s *t* test and 1-way ANOVA were used to compare the means of 2 groups of samples and 3 or more groups of samples, respectively. Tukey’s method was applied to adjust the multiple comparisons. Linear mixed model was used for repeated measurement analysis of tumor growth. The survival difference between the different treatments was assessed by log-rank test. GraphPad Prism 7.03 software was used for statistical analysis. Two-sided *P* values of less than 0.05 were considered statistically significant.

### Study approval.

All mouse experiments were approved by The Ohio State University IACUC. Human melanoma PDOs were established and deidentified previously (23, 28). No new human tissue was collected as a part of this study.

### Data availability.

All data associated with this study are present in the paper or the supplemental materials, and raw data are included in the Supporting Data Values file.

## Author contributions

VB, AK, YW, NR, DDLB, BBK, MC, and RW performed experiments and analyzed data. CGC analyzed data. GH provided reagents, obtained and analyzed data, and advised on study design and data interpretation. AEV conceptualized the study, designed and performed experiments, analyzed data, obtained funding, and supervised the study. AEV, GH, and YW wrote the manuscript. All authors reviewed the manuscript. VB, AK, and YW contributed equally and are co–first authors. The authorship order among co–first authors was assigned using alphabetical order.

## Supplementary Material

Supplemental data

Supplemental table 1

Supplemental table 2

Supplemental table 3

Supporting data values

## Version 1 06/20/2024

In-Press Preview

## Version 2 08/08/2024

Electronic publication

## References

[1] Yamagishi Y (2012). MPS1/Mph1 phosphorylates the kinetochore protein KNL1/Spc7 to recruit SAC components. Nat Cell Biol.

[2] Jemaa M (2013). Characterization of novel MPS1 inhibitors with preclinical anticancer activity. Cell Death Differ.

[3] Schulze VK (2020). Treating cancer by spindle assembly checkpoint abrogation: discovery of two clinical candidates, BAY 1161909 and BAY 1217389, targeting MPS1 kinase. J Med Chem.

[4] RA MS (2022). Development of the first covalent monopolar spindle kinase 1 (MPS1/TTK) inhibitor. J Med Chem.

[5] Chan CY (2022). CFI-402257, a TTK inhibitor, effectively suppresses hepatocellular carcinoma. Proc Natl Acad Sci U S A.

[6] Mason JM (2017). Functional characterization of CFI-402257, a potent and selective Mps1/TTK kinase inhibitor, for the treatment of cancer. Proc Natl Acad Sci U S A.

[7] Schoffski P (2022). First-in-man, first-in-class phase I study with the monopolar spindle 1 kinase inhibitor S81694 administered intravenously in adult patients with advanced, metastatic solid tumours. Eur J Cancer.

[8] Anderhub SJ (2019). High proliferation rate and a compromised spindle assembly checkpoint confers sensitivity to the MPS1 inhibitor BOS172722 in triple-negative breast cancers. Mol Cancer Ther.

[9] Maia ARR (2018). Mps1 inhibitors synergise with low doses of taxanes in promoting tumour cell death by enhancement of errors in cell division. Br J Cancer.

[10] Wengner AM (2016). Novel Mps1 kinase inhibitors with potent antitumor activity. Mol Cancer Ther.

[11] Atrafi F (2021). A phase I study of an MPS1 inhibitor (BAY 1217389) in combination with paclitaxel using a novel randomized continual reassessment method for dose escalation. Clin Cancer Res.

[12] Bedard P (2020). 19O A phase Ib trial of CFI-402257 in combination with weekly paclitaxel in patients with advanced HER2-negative (HER2-) breast cancer (aBC). Ann Oncol.

[13] Bedard P (2023). Abstract P3-07-10: CCTG IND.236: A Phase 1b trial of combined CFI-402257 and weekly paclitaxel in patients with HER2-negative (HER2-) advanced breast cancer (aBC). Cancer Res.

[14] Schöffski P (2022). First-in-man, first-in-class phase I study with the monopolar spindle 1 kinase inhibitor S81694 administered intravenously in adult patients with advanced, metastatic solid tumours. Eur J Cancer.

[15] Mates M (2021). 38MO IND.236: A Canadian Cancer Trial Group (CCTG) phase Ib trial of combined CFI-402257 and weekly paclitaxel (Px) in patients with HER2-negative (HER2-) advanced breast cancer (BC). Ann Oncol.

[16] Wesolowski R (2022). TWT-203: Phase 1b/2 dose-confirming study of CFI-402257 as a single agent in advanced solid tumors and in combination with fulvestrant in patients with ER+/HER2- advanced breast cancer after disease progression on prior CDK4/6 and endocrine therapy. J Clin Oncol.

[17] Sugimoto Y (2017). Novel pyrrolopyrimidines as Mps1/TTK kinase inhibitors for breast cancer. Bioorg Med Chem.

[18] Longo LVG (2024). TTK/MPS1 inhibitor OSU-13 targets the mitotic checkpoint and is a potential therapeutic strategy for myeloma. Haematologica.

[19] Uzhachenko RV (2021). Metabolic modulation by CDK4/6 inhibitor promotes chemokine-mediated recruitment of T cells into mammary tumors. Cell Rep.

[20] Kumar A (2023). Dendritic cell therapy augments antitumor immunity triggered by CDK4/6 inhibition and immune checkpoint blockade by unleashing systemic CD4 T-cell responses. J Immunother Cancer.

[21] Vilgelm AE (2016). Connecting the dots: therapy-induced senescence and a tumor-suppressive immune microenvironment. J Natl Cancer Inst.

[22] Vilgelm AE (2015). Mdm2 and aurora kinase a inhibitors synergize to block melanoma growth by driving apoptosis and immune clearance of tumor cells. Cancer Res.

[23] Bharti V (2022). BCL-xL inhibition potentiates cancer therapies by redirecting the outcome of p53 activation from senescence to apoptosis. Cell Rep.

[24] Coppe JP (2010). The senescence-associated secretory phenotype: the dark side of tumor suppression. Annu Rev Pathol.

[25] Saleh T (2020). Therapy-induced senescence: an “old” friend becomes the enemy. Cancers (Basel).

[26] Sharma P (2023). Immune checkpoint therapy-current perspectives and future directions. Cell.

[27] Vilgelm AE (2016). Combinatorial approach to cancer immunotherapy: strength in numbers. J Leukoc Biol.

[28] Vilgelm AE (2020). Fine-needle aspiration-based patient-derived cancer organoids. iScience.

[29] Schmitt CA (2022). Senescence and cancer - role and therapeutic opportunities. Nat Rev Clin Oncol.

[30] Mackenzie KJ (2017). cGAS surveillance of micronuclei links genome instability to innate immunity. Nature.

[31] Li T, Chen ZJ (2018). The cGAS-cGAMP-STING pathway connects DNA damage to inflammation, senescence, and cancer. J Exp Med.

[32] Landwehr LS (2020). Interplay between glucocorticoids and tumor-infiltrating lymphocytes on the prognosis of adrenocortical carcinoma. J Immunother Cancer.

[33] Lu Y (2014). Tumor-specific IL-9-producing CD8+ Tc9 cells are superior effector than type-I cytotoxic Tc1 cells for adoptive immunotherapy of cancers. Proc Natl Acad Sci U S A.

[34] Purwar R (2012). Robust tumor immunity to melanoma mediated by interleukin-9-producing T cells. Nat Med.

[35] Nonomura Y (2016). Peripheral blood Th9 cells are a possible pharmacodynamic biomarker of nivolumab treatment efficacy in metastatic melanoma patients. Oncoimmunology.

[36] Forget MA (2018). Prospective analysis of adoptive TIL therapy in patients with metastatic melanoma: response, impact of anti-CTLA4, and biomarkers to predict clinical outcome. Clin Cancer Res.

[37] Wang L (2022). Exploiting senescence for the treatment of cancer. Nat Rev Cancer.

[38] Jin Y (2022). Different syngeneic tumors show distinctive intrinsic tumor-immunity and mechanisms of actions (MOA) of anti-PD-1 treatment. Sci Rep.

[39] Vilgelm AE, Richmond A (2019). Chemokines modulate immune surveillance in tumorigenesis, metastasis, and response to immunotherapy. Front Immunol.

[40] Morad G (2021). Hallmarks of response, resistance, and toxicity to immune checkpoint blockade. Cell.

[41] Yost KE (2021). Recruiting T cells in cancer immunotherapy. Science.

[42] Sheppard P (2003). IL-28, IL-29 and their class II cytokine receptor IL-28R. Nat Immunol.

[43] Li Q (2013). Novel type III interferons produce anti-tumor effects through multiple functions. Front Biosci (Landmark Ed).

[44] Silver JS, Hunter CA (2010). gp130 at the nexus of inflammation, autoimmunity, and cancer. J Leukoc Biol.

[45] Wolf J (2016). Different soluble forms of the interleukin-6 family signal transducer gp130 fine-tune the blockade of interleukin-6 trans-signaling. J Biol Chem.

[46] Jostock T (2001). Soluble gp130 is the natural inhibitor of soluble interleukin-6 receptor transsignaling responses. Eur J Biochem.

[47] Johnson DE (2018). Targeting the IL-6/JAK/STAT3 signalling axis in cancer. Nat Rev Clin Oncol.

[48] Yang H (2017). cGAS is essential for cellular senescence. Proc Natl Acad Sci U S A.

[49] Loo TM (2020). Cellular senescence and senescence-associated secretory phenotype via the cGAS-STING signaling pathway in cancer. Cancer Sci.

[50] Li Z (2020). When STING meets viruses: sensing, trafficking and response. Front Immunol.

[51] Kitajima S (2022). MPS1 inhibition primes immunogenicity of KRAS-LKB1 mutant lung cancer. Cancer Cell.

[52] Flynn PJ (2021). Chromatin bridges, not micronuclei, activate cGAS after drug-induced mitotic errors in human cells. Proc Natl Acad Sci U S A.

[53] Konno H (2018). Suppression of STING signaling through epigenetic silencing and missense mutation impedes DNA damage mediated cytokine production. Oncogene.

[54] Falahat R (2021). Epigenetic reprogramming of tumor cell-intrinsic STING function sculpts antigenicity and T cell recognition of melanoma. Proc Natl Acad Sci U S A.

[55] Kwon J, Bakhoum SF (2020). The cytosolic DNA-sensing cGAS-STING pathway in cancer. Cancer Discov.

[56] Hadfield MJ (2023). Stimulator of interferon gene (STING) expression as a biomarker for overall survival in PDL1-negative, TMB-low non-small cell lung cancer (NSCLC) treated with immune checkpoint inhibitors (ICIs). J Clin Oncol.

