# Supplementary material for: TTK inhibitor OSU13 promotes immunotherapy responses by activating tumor STING
Source: JCI Insight. 2024 Jun 20;9(15):e177523. doi: 10.1172/jci.insight.177523 (PMC11383830; doi:10.1172/jci.insight.177523)
Supplement: Supplemental data [file jciinsight-9-177523-s189.pdf]

Supplementary Figures

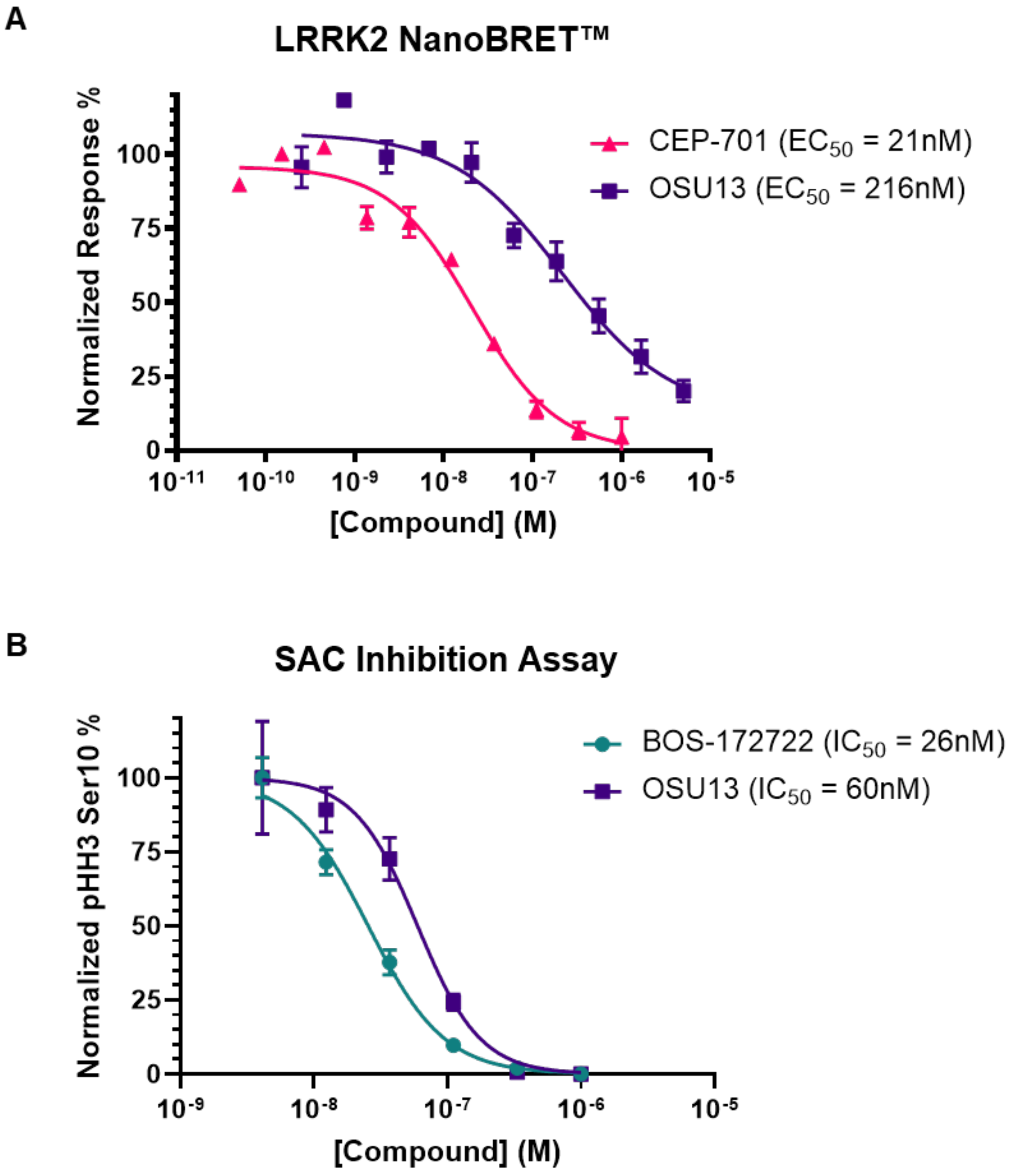

Figure S1. TTK and spindle assembly checkpoint are targeted by OSU13. A. LRRK2 target engagement assay (NanoBRET™) in HEK293 cells. Relative levels of inhibition by OSU13 or the positive control CEP-701 of LRRK2-NanoLuc binding to a fluorescent tracer were measured. Half maximal effective concentrations (EC50) (nM) were calculated. B. Spindle Assembly Checkpoint (SAC) assay in the CAL-51 human breast adenocarcinoma cell line. Relative levels of phosphor-Histone H3 (Ser10) were measured as a function of treatment with a 3-fold dilution series of OSU13 or the reference TTK inhibitor BOS-172722. Half maximal inhibitory concentrations (IC50) (nM) were calculated.

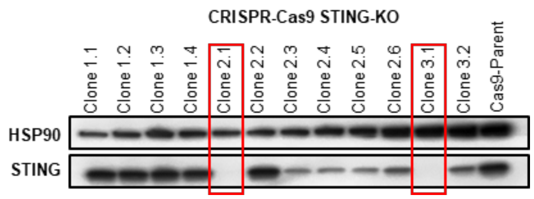

Figure S2. Generation of STING-KO cells. Western blot analysis of STING expression in clones of A375-Cas9 cells after transfection with STING-specific sgRNA and FACS sorting of GFP-positive plasmid-expressing cells.

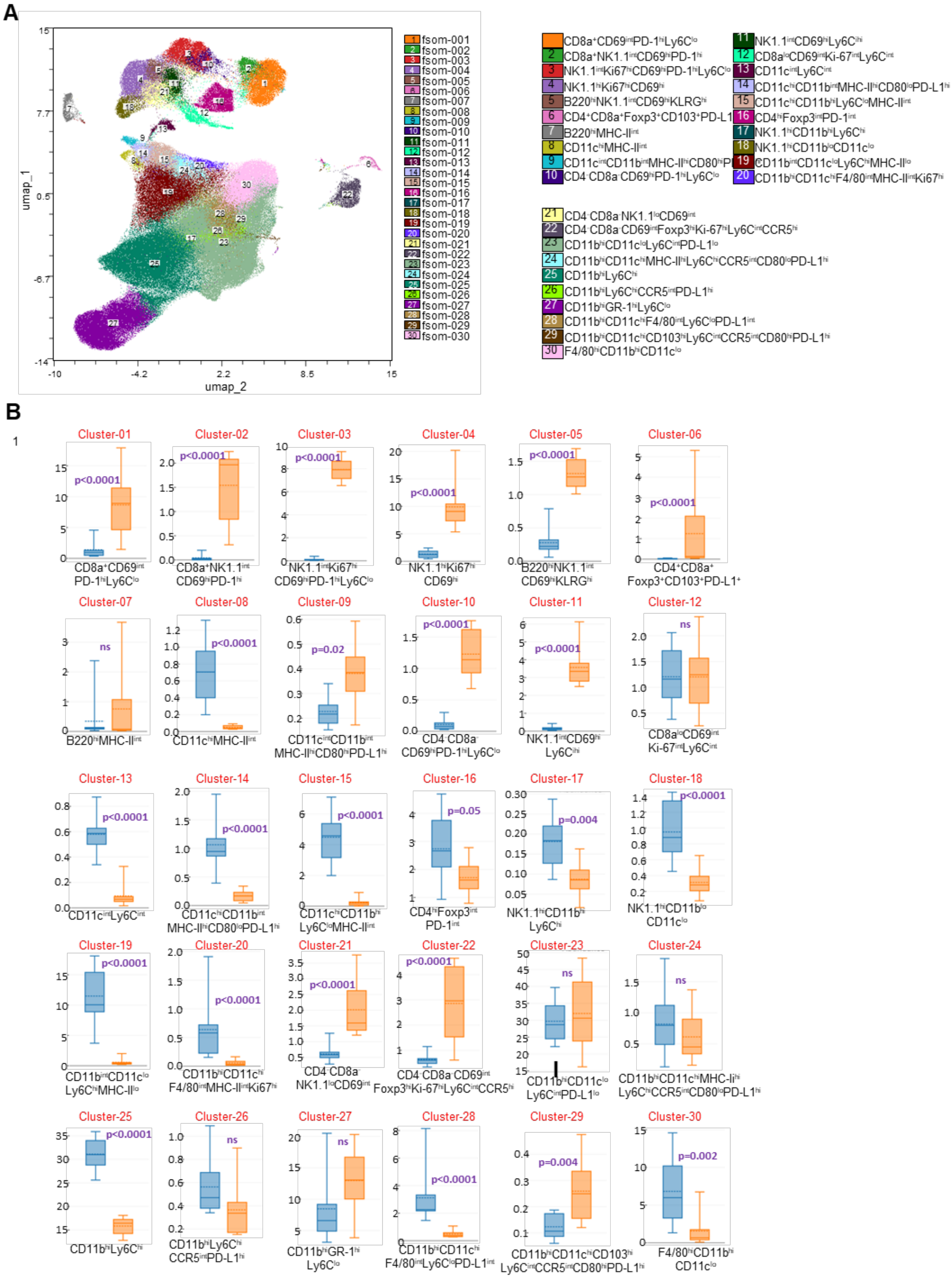

Figure S3. The results of the clustering of spectral cytometry data from vehicle and OSU13-treated tumors. Summary of the distribution and marker expression in 30 cell clusters identified using FlowSOM using spectral cytometry data described in Figure 5D and E. B. The differences in the percentages of CD45+ cells in indicated clusters between vehicle and OSU13-treated tumors. Statistical analysis using edgeR.

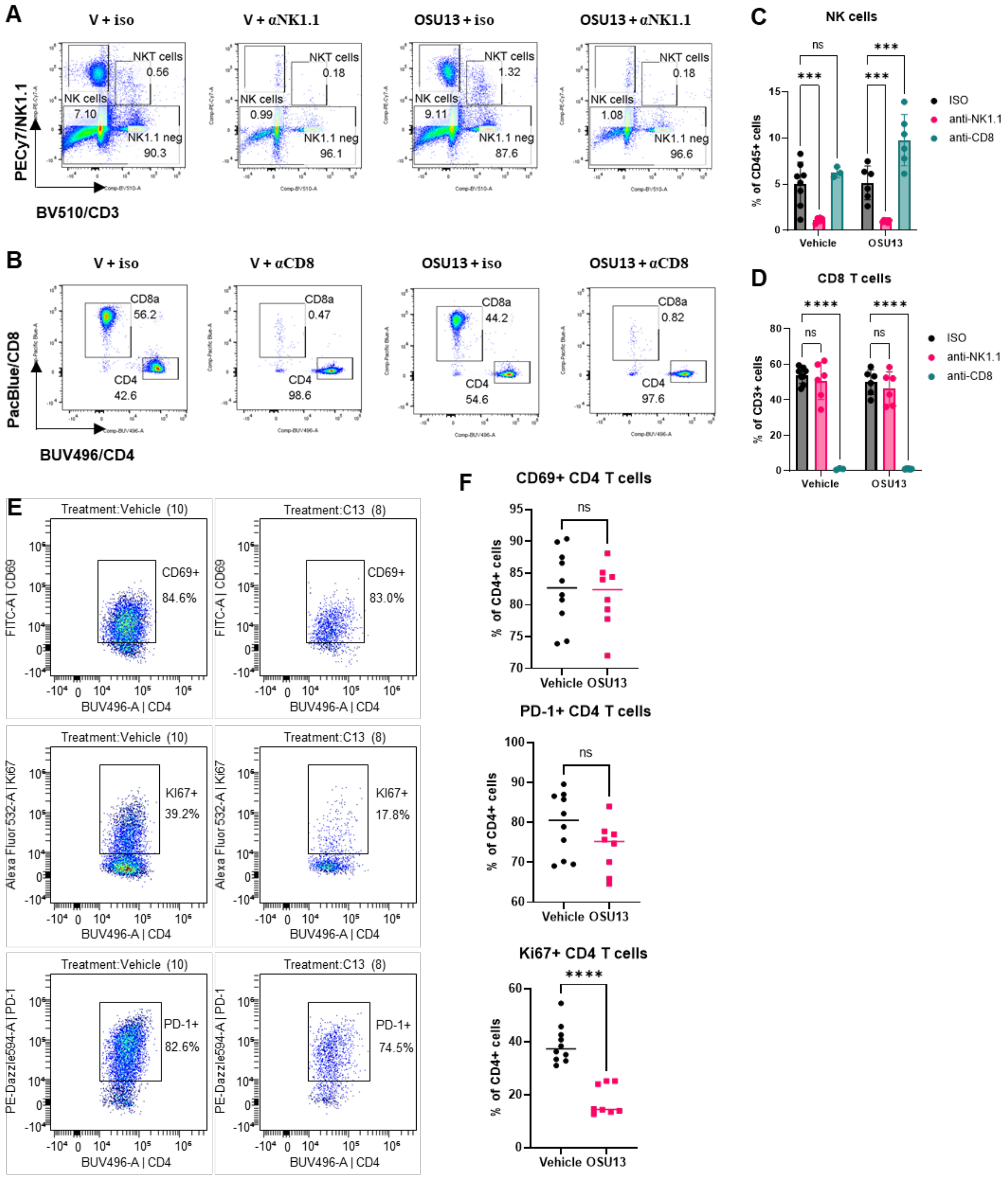

Figure S4. Spectral cytometry analysis of immune infiltrate after OSU13 treatment. A-D. Representative flow cytometry plots (A-B) and quantified data (C-D) demonstrating the efficacy of NK1.1 and CD8 cell depletion in the experiment shown in Fig. 5H. E. Expression of indicated phenotype markers on CD4 T cells in tumors shown in Fig. 5A. F. Quantification of indicated phenotype marker expression in CD4 T cells from tumors shown in Fig. 5A. Statistics using t-test.

A

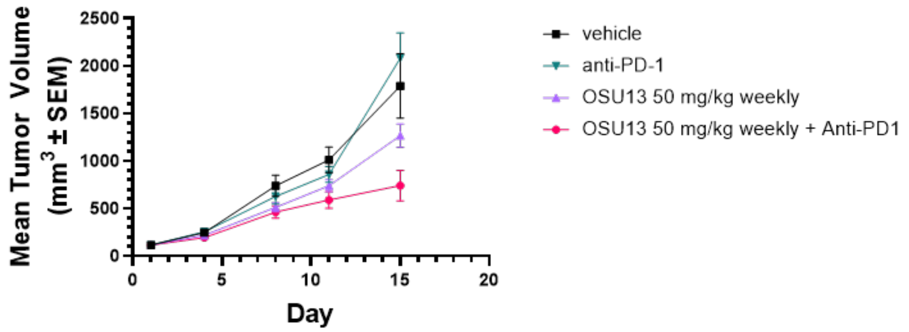

Tukey's multiple comparisons test	Mean Diff.	95.00% CI of diff.	Summary	Adjusted P Value
Vehicle vs. anti-PD1	-81.10	-326.2 to 164.0	ns	0.8274
Vehicle vs. OSU13 50 mg/kg QWK	83.11	-156.5 to 322.8	ns	0.8063
Vehicle vs. OSU13 50 mg/kg, QWK +anti-PD-1	472.3	236.9 to 707.8	****	<0.0001
anti-PD1 vs. OSU13 50 mg/kg QWK	164.2	-77.57 to 406.0	ns	0.2968
anti-PD1 vs. OSU13 50 mg/kg, QWK +anti-PD-1	553.4	313.3 to 793.6	****	<0.0001
OSU13 50 mg/kg QWK vs. OSU13 50 mg/kg, QWK +anti-PD-1	389.2	156.3 to 622.2	***	0.0001

B

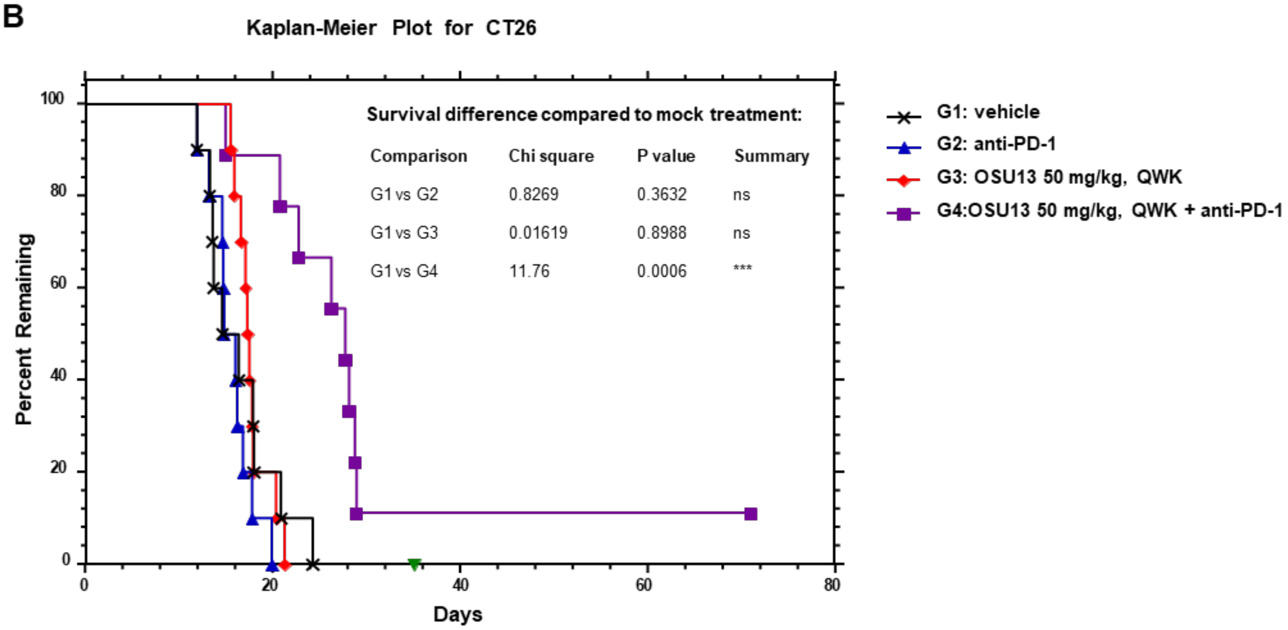

Figure S5. OSU13 promotes anti-PD-1 responses in tumor-bearing mice. A. Tumor growth in BALB/c mice implanted with CT26 tumors and treated with 5 mg/kg anti-PD-1 intraperitoneally biweekly and 50 mg/kg OSU13 weekly by oral gavage. N=10 mice per group. Statistics using mixed model. B. Survival of mice described in A.

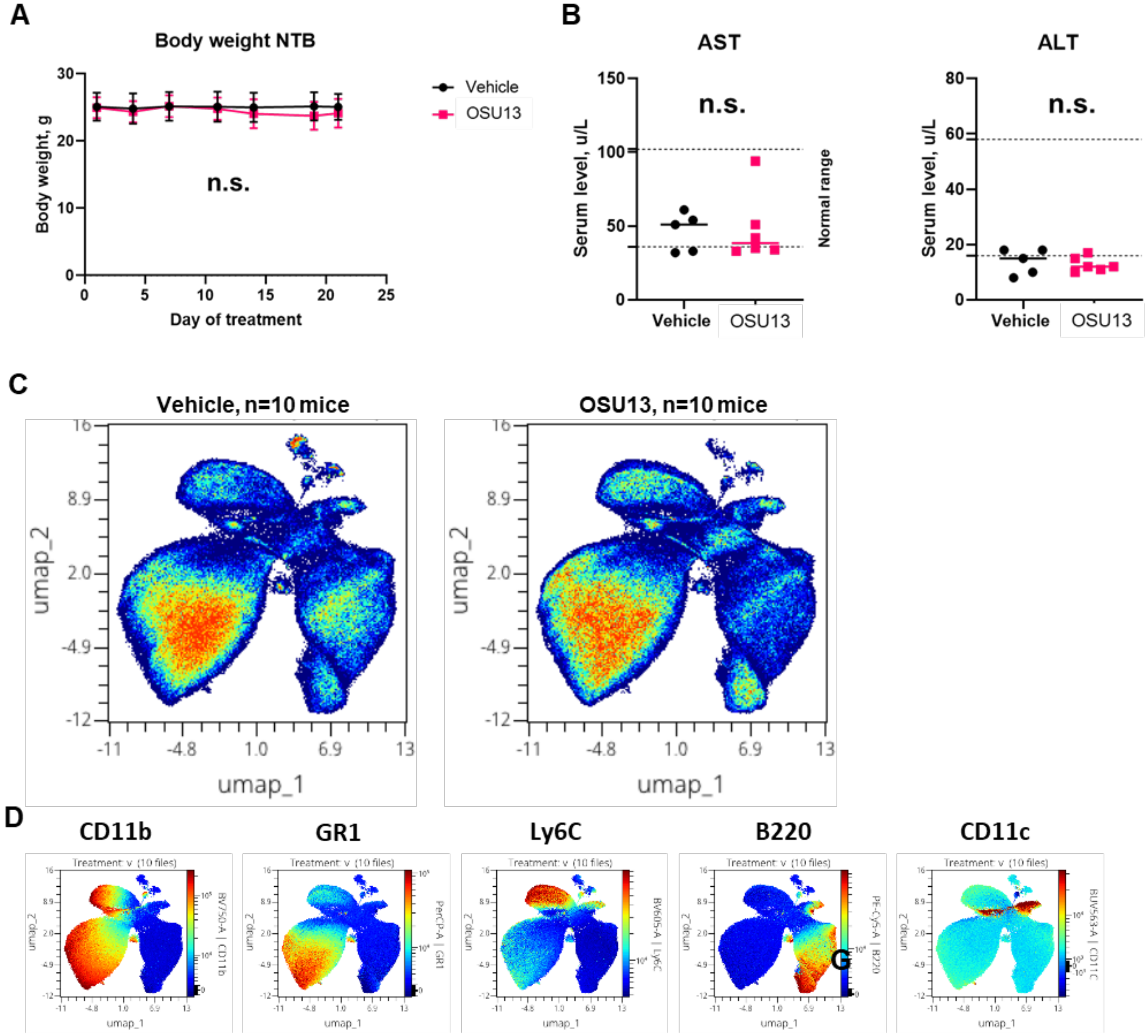

Figure S6. OSU13-treated BALB/c mice display no signs of severe toxicities when treated 5 days a week. A. Changes in body weight over time in tumor-free BALB/c mice that were treated with 10mg/kg OSU13 or vehicle 5 days a week for 3 weeks. B. Analysis of indicated liver proteins in the serum of mice described in A. C-D. Spectral cytometry analysis of bone marrow cells from mice described in A. Dimension reduction analysis and distribution of select immune marker expression (D) are shown.
