# Supplementary material for: TTK inhibitor OSU13 promotes immunotherapy responses by activating tumor STING
Source: JCI Insight. 2024 Jun 20;9(15):e177523. doi: 10.1172/jci.insight.177523 (PMC11383830; doi:10.1172/jci.insight.177523)
Supplement: Supplemental table 3 [file jciinsight-9-177523-s192.docx]

| S.NO. | Antigen | Antibody clone | Fluorochrome | Vendor | Catalog number |
| --- | --- | --- | --- | --- | --- |
| **Antibodies used for MC38 tumor analysis and BM analysis in BALB/c mice** | | | | | |
| 1 | CD45 | 30-F11 | BUV395 | BD Biosciences | 564279 |
| 2 | CD4 | RM4-5 | BUV496 | BD Biosciences | 741050 |
| 3 | CD8 | 53-6.7 | Pacific Blue | Biolegend | 100725 |
| 4 | B220 | RA3-6B2 | PE/Cy5 | Biolegend | 103210 |
| 5 | NK1.1 | PK136 | PE/Cy7 | Biolegend | 108714 |
| 6 | FoxP3 | MF-14 | PE | Biolegend | 126404 |
| 7 | CD3 | 145-2C11 | BV510 | BD Biosciences | 563024 |
| 8 | PD-1 | 29F.1A12 | PE-Dazzle 594 | Biolegend | 135228 |
| 9 | Ki67 | SoIA15 | Alexa Fluor 532 | Invitrogen | 58-5698-82 |
| 10 | CD11b | M1/70 | BV750 | Biolegend | 101267 |
| 11 | CD11c | N418 | BUV563 | BD Biosciences | 749040 |
| 12 | Gr1 | RB6-8C5 | PerCP | Biolegend | 108426 |
| 13 | CD69 | H1.2F3 | FITC | Invitrogen | 11-0691-85 |
| 14 | I-A/I-E | M5/114.15.2 | AF700 | Biolegend | 107622 |
| 15 | KLRG1 | 2F1 | BV480 | BD Biosciences | 746353 |
| 16 | CD103 | M290 | BV786 | BD Biosciences | 564322 |
| 17 | PD-L1 | MIH5 | BV650 | BD Biosciences | 740614 |
| 18 | F4/80 | BM8 | APC | Biolegend | 123116 |
| 19 | CD80 | 16-10A1 | BUV805 | BD Biosciences | 741956 |
| 20 | CCR5 | HM-CCR5 (7A4) | PerCP-eFluor 710 | Invitrogen | 46-1951-82 |
| 21 | Ly6C | HK1.4 | BV605 | Biolegend | 128035 |
| 22 | Viability | NA | efluor780 | Invitrogen | 50-112-9035 |
| **Antibodies used for BM analysis in C57BL/6 mice** | | | | | |
| 1 | CD45 | 30-F11 | BUV395 | BD Biosciences | 564279 |
| 2 | CD4 | RM4-5 | BUV496 | BD Biosciences | 741050 |
| 3 | CD8 | 53-6.7 | Pacific Blue | Biolegend | 100725 |
| 4 | B220 | RA3-6B2 | PE/Cy5 | Biolegend | 103210 |
| 5 | NK1.1 | PK136 | PE/Cy7 | Biolegend | 108714 |
| 6 | FoxP3 | MF-14 | PE | Biolegend | 126404 |
| 7 | CD3 | 17A2 | PerCP-eFluor 710 | Invitrogen | 46-0032-82 |
| 8 | PD-1 | 29F.1A12 | PE-Dazzle 594 | Biolegend | 135228 |
| 9 | Ki67 | SoIA15 | Alexa Fluor 532 | Invitrogen | 58-5698-82 |
| 10 | CD11b | M1/70 | BV750 | Biolegend | 101267 |
| 11 | CD11c | N418 | BUV563 | BD Biosciences | 749040 |
| 12 | Gr1 | RB6-8C5 | PerCP | Biolegend | 108426 |
| 13 | I-A/I-E | M5/114.15.2 | AF700 | Biolegend | 107622 |
| 14 | PD-L1 | MIH5 | BV650 | BD Biosciences | 740614 |
| 15 | F4/80 | BM8 | APC | Biolegend | 123116 |
| 16 | Ly6C | HK1.4 | BV605 | Biolegend | 128035 |
| 17 | Viability | NA | efluor780 | Invitrogen | 50-112-9035 |
